# Development of Measures of Perceived Neighborhood Environmental Attributes Influencing, and Perceived Barriers to Engagement in, Healthy Behaviors for Older Chinese Immigrants to Australia

**DOI:** 10.3390/ijerph18094531

**Published:** 2021-04-24

**Authors:** Ester Cerin, Shiyuan Yin, Wing Ka Choi, Winsfred Ngan, Rachel Tham, Anthony Barnett

**Affiliations:** 1 Mary MacKillop Institute for Health Research, Australian Catholic University, Melbourne, VIC 3000, Australia; Kako.Choi@acu.edu.au (W.K.C.); Rachel.Tham@acu.edu.au (R.T.); Anthony.Barnett@acu.edu.au (A.B.); 2 School of Public Health, The University of Hong Kong, Pokfulam, Hong Kong, China; 3 Baker Heart and Diabetes Institute, Melbourne, VIC 3004, Australia; 4 School of Exercise and Nutrition Science, Deakin University, Burwood, VIC 3125, Australia; gwyinshiyuan@gmail.com (S.Y.); wwtngan@gmail.com (W.N.)

**Keywords:** walkability, food environment, immigrants, aging, socializing, neighborhood, Australia

## Abstract

Environmental correlates, barriers, and facilitators of physical activity, healthy eating, and socializing are understudied in older immigrants to developed countries. This study developed/adapted and validated measures of perceived barriers and neighborhood environmental characteristics related to these health-enhancing behaviors appropriate for older Chinese immigrants to Australia and similar Western countries. Older Chinese immigrants living in Melbourne (Australia) were recruited from neighborhoods varying in walkability and percentage of Chinese residents. Versions of the Neighborhood Environment for Healthy Aging–Chinese Immigrants to Australia (NEHA-CIA) questionnaire (20 subscales) and the Perceived Barriers to Health-Enhancing Behaviors questionnaire (four subscales) were developed from extant validated scales and information collected in formative qualitative research. Thirty-one participants took part in cognitive interviews aimed to pilot-test and refine the questionnaires. The modified questionnaires were administered to 52 participants twice, two weeks apart. Test-retest reliability (intraclass correlation coefficients), internal consistency (Cronbach’s α), and construct validity (associations with theoretically-relevant constructs) were examined. Most items and subscales of both questionnaires had good test-retest reliability and internal consistency, while the NEHA-CIA also showed good construct validity. Future studies need to further examine the construct validity of the questionnaire of perceived barriers and determine the factorial validity of both measures on large representative samples.

## 1. Introduction

Physical activity, healthy eating, and socializing are key contributors to healthy aging. Regular engagement in physical activity and a healthy Mediterranean diet have been associated with a reduced risk of premature death [1,2] and major chronic diseases in late life including cancer [2,3], cardiovascular diseases [2,4], type 2 diabetes [5,6], and dementia [2,7]. Additionally, older adults lacking social contacts tend to have difficulties recovering from life threatening events [8] and be at increased risk of depression [9], cognitive decline [7], and premature death [10].

As with any health-enhancing behavior, physical activity, healthy eating, and social engagement are shaped by a multitude of factors including socio-demographic characteristics (e.g., ethnicity and sex), psychosocial factors (e.g., self-efficacy and enjoyment in the behavior), and the social and physical attributes of one’s proximal (e.g., household) and distal environments (e.g., community) [11]. Over the last 15 years, the neighborhood environment, in particular, has been identified as an important source of influences on older adults’ health-related behaviors [12,13,14,15] and health [16,17,18,19]. This is because older people’s reduced mobility and physical functionality make them more vulnerable to unfavorable environmental conditions and more reliant on their local environment [17]. The impact of the neighborhood environment is likely to be even greater for older immigrants due to post-migration cultural differences and poor language proficiency limiting their independence and mobility [20]. However, research on the effects of the neighborhood environment on older immigrants’ health-related behaviors such as physical activity, healthy eating, and social engagement is lacking [20,21]. The dearth of research in this demographic group is at odds with global demographic trends reporting over 12 million more older immigrants in developed regions in 2019 than there were in 1990 [22].

To examine the effects of the neighborhood environment on health-enhancing behaviors, appropriate exposure measures that capture sentinel aspects of the environment that are relevant to a specific behavior, population, and geographical context are needed. Environmental attributes are usually assessed using archival datasets analyzed with geographic information systems software, systematic observations (environmental audits) and questionnaires. Whilst the first two methods provide objective data on the environment, questionnaires are used to collect data on the residents’ perceptions of their environment. Each of these methods offers equally important complementary data that contribute to the understanding of how the environment influences the behavior in question [23,24]. Collecting information on perceived aspects of the neighborhood environment via questionnaires is important because individuals differ in their perceptions and evaluations of the same objective environment [23,24]. Additionally, people’s perceptions of the environment are generally more strongly related to their behaviors than are objective measures of the environment [12,24].

With a few exceptions (e.g., [25,26]), questionnaires gauging environmental attributes relevant to health-enhancing behaviors such as physical activity have been developed for mainstream populations across various countries [27,28,29] or specific geographical contexts [30,31,32] and then applied to minority and immigrant populations [33,34,35,36]. This approach has advantages as well as disadvantages. Whilst it is important and useful to employ measures that allow for a comparison of findings across different ethnic and socio-demographic groups [27], it is also necessary to acknowledge that minorities and immigrant populations may face unique challenges (e.g., language barriers [20,21]) and have specific cultural needs (e.g., culturally-appropriate forms of physical activity [37]) that are not captured by measures developed for the mainstream population and end up being overlooked and left unaddressed [38]. Therefore, the development of questionnaires gauging immigrants’ perceptions of universally important as well culturally and context-relevant environmental characteristics impacting health-enhancing behaviors is an essential step toward the development of this research field.

This study aimed to contribute to addressing the lack of measures of perceived neighborhood environmental attributes influencing older immigrants’ health-enhancing behaviors by developing working versions of questionnaires appropriate for older Chinese immigrants to Australia (1) capturing aspects of the neighborhood environment potentially important for the promotion of physical activity, social engagement, and healthy eating, and (2) assessing perceived individual, social, and environmental barriers to engagement in the same behaviors. We focused on the development of measures for older Chinese immigrants to Australia because Australia is one of the developed regions with the largest increases in older immigrants worldwide [39] and Chinese immigrants are the largest minority group of non-English-speaking background in Australia [40]. Furthermore, we aimed to develop measures targeting physical activity, healthy eating, and socializing because studies suggest that the promotion of these behaviors is important for the well-being of this population. Specifically, older Chinese immigrants to Australia have been found to be less physically active than non-immigrants [41], even though older Chinese living in their country of birth are typically much more active than their Australian counterparts [42,43,44]. A similar pattern of findings has been observed with respect to fruit and vegetable consumption (i.e., healthy eating) [41,45]. There is also evidence that older Chinese immigrants to Western countries often suffer from depression because their age, low level of language proficiency, cultural barriers, and relocation to an unfamiliar environment lead to social isolation [46,47,48]. In developing the aforementioned questionnaires, we aimed to capture issues and factors that are idiosyncratic to older Chinese immigrants to Australia as well as factors that are relevant to other socio-demographic groups and geographical contexts to facilitate cross-cultural comparisons.

## 2. Materials and Methods

This study consisted of a qualitative and a quantitative stage. This study consisted of a qualitative and a quantitative stage. The qualitative stage had two components: (a) development of a list of items for each questionnaire based on extant validated questionnaires, published findings from NGT sessions [21], and expert panel knowledge; and (b) refinement of questionnaires based on feedback obtained from cognitive interviews. In the quantitative stage, item and (sub)scale test-retest reliability and, where appropriate, (sub)scale internal consistency were examined. The study was approved by the Deakin University Human Ethics Advisory Group, Faculty of Health (ref. no: 161-2014; 9 October 2014).

### 2.1. Participants

We recruited three purposive convenience samples of older Chinese immigrants (60+ years) from administrative units (Statistical Areas Level 1; SA1s) in Melbourne (Australia) varying in walkability and percentage of residents of Chinese background. This type of stratified sampling strategy aimed to maximize the heterogeneity of the sample in terms of levels of exposure to amenities (impacting on physical activity) [12,13], availability of Chinese ethnic foods, and opportunities to socialize with Chinese-speaking residents. Geographic information systems (GIS) were used to first stratify SA1s into high (fourth quartile) and low (first quartile) walkable areas based on a composite index representing the sum of SA1s’ standardized scores of dwelling density, street intersection density, and land use mix [43,49,50]. Using census data on ethnic composition, high- and low-walkable SA1s were subsequently classified into areas with medium (5–10%) or high (>10%) prevalence of Chinese residents to obtain four types of neighborhood: high walkable/high Chinese prevalence (HW/HC), high walkable/medium Chinese prevalence (HW/MC), low walkable/high Chinese prevalence (LW/HC), and low walkable/medium Chinese prevalence (LW/MC) [21]. The selected SA1s were low-to-medium income with an Index of Relative Advantage and Disadvantage (IRSAD) ranging from 4 to 7 out of 10 (70% with an IRSAD of 5 or 6).

Participants were eligible if they were born overseas and ethnically Chinese, aged 60+ years, able to speak and read Chinese, cognitively intact (i.e., with no diagnosis of mild cognitive impairment or dementia, and no significant perceived memory problems), able to walk unassisted and resided in one of the pre-selected SA1s. Recruitment was conducted via several channels including advertisements in Chinese newspapers, presentations, and posters in community centers, churches, medical centers, and libraries. All recruitment material was available in traditional and simplified Chinese as well as in English. Potential participants were invited to contact research staff (via email or phone) speaking their preferred language (Cantonese, Mandarin, or English) to obtain further information about the study and for eligibility assessment. Eligible participants provided written informed consent prior to participating in the study. Ninety-one participants took part in the nominal group technique (NGT) sessions [21] that informed the content of questionnaire items, 31 in the cognitive interviews, and 52 in the quantitative stage of the study. Table 1 shows the socio-demographic characteristics of the participants in the latter two stages of the study, while the characteristics of the participants in the NGT sessions have been reported elsewhere [21]. Sexes, various age groups, and neighborhood types were well represented in both samples. The majority of participants were Mandarin-speakers and living with adult children. A substantial proportion of them migrated to Australia within five years prior to participating in this study.

### 2.2. Procedures and Instruments

#### 2.2.1. Qualitative Stage—Questionnaire Development and Translation

The aim of this study was to develop a self-administered questionnaire assessing perceived neighborhood environmental attributes related to physical activity, social engagement, and healthy eating (hereafter named ‘Neighborhood Environment for Healthy Aging-Chinese Immigrants to Australia’, NEHA-CIA), and self-administered questionnaires assessing perceived individual, social, and environmental barriers to engagement in a specific behavior (physical activity, healthy eating, or social engagement) appropriate for older Chinese immigrants to Australia. Two sources were used to generate a pool of potential items for these questionnaires: (1) expert panel (research team) knowledge on relevant extant published and unpublished questionnaires and studies; and (2) statements collected in a formative qualitative study of older Chinese immigrants to Australia (NGT component of this project presented elsewhere) on built and social environmental facilitators and barriers to engagement in physical activity, healthy eating, and socializing [21].

The questionnaires were developed in English, translated into simplified and traditional Chinese by two bilingual staff members (if Chinese versions of selected items were not available), and back-translated in English by an independent bilingual researcher. Discrepancies between the original and back-translated versions were reviewed and resolved by the research team and translators. The content of the questionnaires is presented in Table 2 and Table 3 together with the sources of the items, relevance for a specific behavior, and modifications made by the panel of experts (if any) prior to administering the questionnaires to study participants in cognitive interviews.

The working version of the NEHA-CIA included subscales/items from the Neighborhood Environment Walkability Scale for Chinese Seniors (NEWS-CS) [32], which assesses aspects of the neighborhood environment related to physical activity relevant to older Chinese and is based on an extensively validated questionnaire (NEWS [27]) used in international studies. NEWS-CS subscales that were selected for inclusion in the NEHA-CIA were: (1) residential density; (2) land use mix-diversity (with minor modifications to the content as explained in Table 2); (3) access to services, which was re-named general access to services to distinguish it from subscales assessing access to specific destinations (with minor modifications); (4) physical barriers to walking; (5) street connectivity; (6) infrastructure for walking, which was re-named infrastructure for pedestrians and to which several single-item scales of the NEWS-CS were added (see Table 2); (7) indoor places for walking; (8) aesthetics; (9) traffic and road hazards; (10) traffic speed; (11) social disorder/littering; (12) crime; and (13) safety—presence of people. All items of the NEWS-CS, except for the residential density and land use diversity subscales, were assessed on a 4-point Likert scale ranging from *strongly disagree* to *strongly agree*. The land use diversity items were rated on a 5-point scale denoting the walking time needed to reach the nearest destination of a certain type (e.g., bakery) from home, with responses ranging from *1- to 5-min* to *>30-min* walking distance. Residential density items gauging the prevalence of various types of residential buildings were rated on a 5-point scale ranging from *none* to *all*. Ratings were weighted relative to the average residential density that a specific item (residential building type) represented. The weighted ratings were summed to obtain a total score.

Subscales from the NEWS-CS were included in the NEHA–CIA not only because they assess factors related to physical activity, but also because some of these subscales/items are potentially relevant to healthy eating [51,52,53] and socializing [54,55,56] (Table 2). For example, the residential density subscale was included because residential density promotes active transportation [12] and may impact on social engagement by providing more opportunities to interact with others [57,58]. Furthermore, in the Australian context, high-density neighborhoods with access to commercial services tend to have better access to healthy food outlets [59]. Hence, in Australia, perceived residential density may also be positively associated with healthy eating. Perceived proximity of various types of destinations from home (land use mix-diversity) and access to services are also strong predictors of older adults’ active transport [12], overall physical activity [21,58] and socializing [21], while food outlet proximity (e.g., fresh food market, supermarket, fast food restaurants) promotes a healthy diet [51].

Findings from NGT sessions on barriers and facilitators of health-enhancing behaviors in older Chinese immigrants to Australia [21] were used to develop several NEHA-CIA subscales gauging behavior-relevant aspects of the environment not captured by the NEWS-CS (Table 2). These included the following subscales: recreational facilities; physical food environment; destinations for socializing; transportation; and a social environment and communication subscale for each of the three behaviors. An item (“It is easy to walk to a transit stop (bus, tram) from home”) from the NEWS-CS was also included in the transportation subscale and a couple of items from the Multi-Ethnic Study of Atherosclerosis (MESA) [26] were included in the physical food environment subscale (“A large selection of low-fat products are available in my neighborhood” and “The fresh fruits and vegetables in my neighborhood are of high quality”). The 4-point Likert scale from the NEWS-CS was used to rate the items of the newly developed subscales of the NEHA-CIA.

As to perceived barriers to engaging in specific behaviors, the expert panel identified potential scales or items describing barriers to physical activity, healthy eating, and socializing employed in studies on older Chinese [60,61] and other populations [62,63,64,65,66,67,68], and complemented them with information gathered in the relevant NGT sessions as described in Table 3. The items were rated on a 5-point scale ranging from *never* to *very often* and indicated how often participants experienced a specific factor to be a barrier to engagement in the specific behavior (physical activity, healthy eating, or socializing).

#### 2.2.2. Qualitative Stage—Cognitive Interviews

The preliminary versions of the questionnaires described above were administered to 31 participants taking part in standardized cognitive interviews (duration: ~60–90 min). Cognitive interviews were conducted to understand the participants’ interpretation of the questionnaire items and the way they decided to give a specific answer, and to establish whether they could match their internally generated answers to the available response categories [69,70]. After providing written informed consent, participants were randomly assigned to one of the two questionnaires (NEHA-CIA or Perceived Barriers to Health Enhancing Behaviors). Sixteen participants completed the NEHA-CIA, while 15 participants completed the Perceived Barriers questionnaire. Participants were asked to verbalize their thought process while they were providing responses to the items. They were also asked to explain the meaning of the items, response options, and how they selected their answers to specific statements, and to identify difficult, unclear, or inappropriate words, statements, or instructions. Finally, they were invited to identify any relevant aspect of the neighborhood environment or barrier to engagement in a specific health-enhancing behavior that was not included in the questionnaires. Interviews were audio-recorded and transcribed verbatim. The findings from the cognitive interviews were used to modify the questionnaires prior to implementing them in the quantitative stage of the study.

#### 2.2.3. Quantitative Stage—Test-Retest Reliability and Construct Validity

A quantitative study was conducted to assess the test-retest reliability, internal consistency, and preliminary construct validity of the working versions of the NEHA-CIA and perceived barriers subscales. Fifty-two participants were recruited for this stage of the study as detailed above. They were asked to self-complete a paper-and-pencil socio-demographic questionnaire and the newly developed questionnaires. The latter were administered again two weeks after the completion of the first survey.

### 2.3. Data Analyses

Test-retest reliability of each item and subscale of the two questionnaires was estimated using intra-class correlation coefficients (ICC) based on absolute agreement two-way mixed effects models. To interpret the results of the ICCs, we used ratings in the following categories: <0.20 (poor); 0.21–0.40 (fair); 0.40–0.59 (moderate); 0.60–0.79 (substantial); and ≥0.80 (excellent) [71]. We also computed percent agreement for items and subscales with restricted variability (in this case, SD <0.5) and ICC <0.60 because low variability may yield low ICC values even when the actual agreement between measurements is high [32]. Items and subscales with restricted variability, low-to-moderate ICC values, and percent agreement >75% were considered highly reliable, and those with percent agreement 60–74% and low ICC values were considered moderately reliable [32]. To establish whether there was a significant systematic change in subscale scores from the first to the second assessment, paired *t*-tests were computed. When appropriate, that is, when the scale was supposed to represent a unidimensional construct rather than an index [32], the internal consistencies of the subscales were estimated using Cronbach’s α coefficient.

To examine the construct (convergent) validity of the NEHA-CIA subscales, we examined the associations between scores at the first assessments with the categorical (dichotomous) measures of area-level walkability (low vs. high) and/or prevalence of Chinese residents (medium vs. high). These associations were estimated using generalized linear models. The subscales measuring residential density, street connectivity and access to shops, transportation, and food outlets were expected to be positively related with area-level walkability (i.e., their scores would be higher in high- than low-walkable areas) because these characteristics are components of the walkability index used to select study areas [31,72]. Items and scales measuring the availability of services in Chinese (including ethnic Chinese foods) and the presence of Chinese-speaking residents and staff were expected to be related to area-level prevalence of Chinese residents. The construct validity of the perceived barriers to health-enhancing behaviors subscales was examined by estimating the association of scores at the first assessment with the scores on theoretically relevant NEHA-CIA subscales (e.g., perceived barriers to healthy eating and physical food environment) and objective environmental measures (binary indicators of walkability and percentage of Chinese residents). All models were adjusted for participant age and sex. Models of associations between perceived barriers to health-enhancing behaviors and perceived environmental attributes (NEHA-CIA subscales) were estimated with adjustment for length of residence in Australia because length of residence influence both sets of variables [12,13].

## 3. Results

### 3.1. Cognitive Interviews

Findings from the cognitive interviews led to several modifications of both NEHA-CIA and perceived barriers subscales, as evidenced in Table 4 and detailed in Table S1. Participants needed to be reassured that there were no right or wrong answers and reminded that the questionnaire was about the current neighborhood in the host country (Australia) rather than China. Most participants indicated that they needed to be often reminded of the researchers’ definition of neighborhood (e.g., in each or most items) and noted that the meaning of the original anchors “somewhat agree” and “somewhat disagree” were unclear to them. They preferred having to provide a more definite answer (i.e., “disagree” and “agree”). They also found it difficult to provide a response on the provided 4-point Likert scale to items that described features that were not present in the neighborhood. For example, for items such as “Chinese clubs have limited resources and/or membership places”, they suggested that a “not applicable” response option be added. They also suggested that a neutral response option (“unsure”) be added. While results from the cognitive interviews led to the addition of only one new item to the NEHA-CIA questionnaire (Table S1), a substantial number of items (27) were modified to enhance clarity by rewording them or providing an explanation for unclear terms as detailed in Table S1 (Supplementary Materials).

### 3.2. Test-Retest Reliability and Internal Consistency

To enable comparison with studies that examined the reliability and validity of the NEWS-CS [32], scores on the NEHA-CIA items rated on 5-point Likert scales were recoded to correspond to the original 4-point Likert scale [1 (strongly disagree) = 1 (strongly disagree); 2 (disagree) = 2 (somewhat disagree); 3 (unsure) = 2.5; 4 (agree) = 3 (somewhat agree); 5 (strongly agree) = 4 (strongly agree)]. Table 5 reports the findings of the test-retest reliability analyses for each subscale of the NEHA-CIA and the Perceived Barriers to Health-Enhancing Behaviors questionnaire, while item-level test-retest reliability analyses are reported in Table S2. The reliability of the NEHA-CIA subscales ranged from moderate (ICC = 0.59; social environment and communication—socializing) to excellent (ICC = 0.97; residential density), while the perceived barriers questionnaire showed substantial reliability with ICCs ranging from 0.70 to 0.77. Significantly different average scores across assessments were observed only for three NEHA-CIA subscales (land use diversity; physical barriers to walking; and traffic and road hazards), whereby more positive evaluations at the second assessment were observed for the latter two subscales.

At the item-level, test-retest reliability was more variable and ranged from fair (ICC = 0.32; item “Parked cars block the vision and make it difficult to cross the road”) to excellent (ICC = 1.00; item “Apartments with >20 storys) for the NEHA-CIA, and from moderate (ICC = 0.45; item “Lack of children’s support for socializing”) to excellent (ICC = 0.91; item “Being independent of other family members”) for the perceived barriers questionnaire (Table S2). Several items of the land use diversity subscale had significantly different average scores at the two assessments and also lower levels of test-retest reliability (fair to moderate). Most of the land use diversity items with significantly different average scores across assessments tended to suggest that participants perceived shorter distances to specific destinations (from home) at the second than the first assessment (Table S2). Items (6 items) from other subscales of the NEHA-CIA with significantly different average scores tended to show more favorable perceptions at the second assessment (e.g., clear information on next train stop). Four NEHA-CIA items had inadequate (i.e., fair) levels of test-retest reliability (Table S2). These were “Distance to the nearest swimming pool” and “Distance to the nearest public toilet” (land use diversity subscale), “Shops in the neighborhood selling fresh fruit and vegetable juice” (physical food environment subscale), and “Parked cars block vision and make it difficult to cross the road” (traffic and road hazards subscale). Deletions of these items from the relevant subscales did not lead to a significant improvement in the test-retest reliability of the subscales.

Only three items of the perceived barriers questionnaires showed significant between-assessment differences in average scores. These were “Difficulties in getting to/from recreational destinations” (lower scores in the second assessment), “Lack of time to walk for transport” (higher scores in the second assessment), and “Lack of enjoyment of healthy foods” (lowers scores in the second assessment). Deletions of these items from the relevant subscales did not lead to a significant improvement in test-retest reliability of the subscales.

All subscales of the perceived barriers questionnaire and most of the subscales of the NEHA-CIA had adequate levels of internal consistency (Cronbach’s α ≥ 0.70) (Table 5). Somewhat lower than acceptable levels of internal consistency were observed for the NEHA-CIA’s transportation, physical barriers to walking, traffic speed, social disorder/littering, and social environment and communication—healthy eating subscales. However, most of these subscales had a small number of items (two or three).

### 3.3. Construct Validity

Table 6 reports the results of the analyses addressing the construct validity of the NEHA-CIA and Perceived Barriers to Health-Enhancing Behaviors questionnaire. As expected, scores on the land use diversity (proximity of destinations), general access to services, transportation, and street connectivity subscales were higher in more walkable neighborhoods. However, contrary to expectations, residential density did not significantly differ between low and high walkable neighborhoods. Other perceived characteristics that were found to be statistically significantly higher in more walkable neighborhoods were infrastructure for pedestrians, traffic and road hazards, and presence of people. Weaker positive associations of physical food environment, indoor places for walking, and social environment and communication—healthy eating with neighborhood walkability were also observed. Positive associations were found between the prevalence of Chinese residents in a neighborhood and the following subscales of perceived neighborhood characteristics: physical food environment, destinations for socializing, presence of people, social environment and communication—Healthy eating and social environment and communication—Socializing.

Fewer significant associations were found between the Perceived Barriers to Health-Enhancing Behaviors subscales and objective and/or perceived characteristics of the neighborhood environment that were deemed to be potentially related to perceived barriers to specific behaviors (Table 6). Specifically, perceived barriers to leisure-time physical activity were significantly negatively related to perceived access to recreational facilities (Recreational facilities subscale of the NEHA-CIA), while perceived barriers to healthy eating and to socializing showed weak negative associations with the physical food environment and social environment and communication—socializing subscales, respectively.

## 4. Discussion

Current trends and future projected increases in migration of aging populations to developed countries [22] demand an investigation into the barriers and facilitators to healthy aging faced by older immigrants. Among the various barriers and facilitators, attributes of the neighborhood environment in the host country are of particular importance not only because they may substantially differ from those of the home country, but also because older immigrants are typically less mobile and more dependent on their local community [20]. To examine these issues, culturally tailored, valid, and reliable measures of factors impacting on older immigrants’ health-enhancing behaviors such as physical activity, healthy eating, and socializing are needed. Hence, this study developed and examined the metric characteristics of self-reported measures of neighborhood environmental attributes and perceived barriers related to health-enhancing behaviors appropriate for older Chinese immigrants to Australia.

### 4.1. Questionnaire Development and Adaptation

The newly-developed measures were based on extant validated questionnaires (e.g., NEWS-CS [32] and perceived barriers to physical activity [61,62]) as well as in-depth qualitative information provided by representatives of the target population [21]. However, while the extant questionnaires previously used in older Chinese samples were interviewer-administered and tailored to Cantonese-speaking older adults, those developed in this study were self-administered. Since there are significant differences between spoken Cantonese (a dialect spoken in Hong Kong) and written Chinese, the interviewer-administered versions of the NEWS-CS and perceived barriers questionnaire needed to be adapted into written Chinese self-administered versions. Cognitive interviews suggested that the newly-developed/adapted self-administered questionnaires were more challenging to complete than the original interviewer-administered versions [61] and required several amendments to ensure that participants understood the questionnaire items and response options. With regard to the NEHA-CIA, it is interesting that participants deemed it necessary to be frequently reminded of the definition of neighborhood (10–15 min walking distance from home), the meaning of the response options “agree” (agree that the statement is true) and “disagree” (do not think that the statement is true), and that the items referred to their host (Australia) rather than home country. This was not necessary for the interviewer-administered version of the NEWS-CS [32], from which the NEHA-CIA was derived, probably because participants could ask questions during the interview and interviewers were able to provide further clarifications if they perceived that the participants misunderstood the instructions or items.

Of note is also the fact that participants suggested that, in the self-administered NEHA-CIA, the originally proposed 4-point Likert response scale be replaced by a 5-point Likert scale with a neutral (“unsure”) response option in the middle of the scale, and that the word “somewhat” be omitted from the anchors “somewhat agree” and “somewhat disagree”. Participants thought that the latter response options were unclear as they implied a certain level of indecisiveness. Similar concerns were raised by members of an expert panel who contributed to the development of the NEWS-CS [32]. However, as a pilot study of the NEWS-CS indicated that the omission of “somewhat” from the anchors might reduce the variability of responses, the developers of the NEWS-CS decided to use the originally proposed 4-point Likert scale with the qualifier “somewhat”. Using the qualifier “somewhat” was not considered problematic because the NEWS-CS was interviewer-administered and participants could seek clarifications during the interview.

The questionnaires developed in the present study include many items that capture aspects of the neighborhood environment, barriers, and facilitators that are relevant to any population of (not only) older adults (e.g., lack of health, time, access to services), some that are particularly relevant to older immigrants in general and others that are idiosyncratic of older Chinese immigrants to Western English-speaking countries. For example, the NEHA-CIA subscale gauging perceived public transport convenience, affordability, and ease of use is bound to be particularly relevant to most groups of older migrants from developing to developed countries because they are less likely to drive [73,74,75] and often hesitate to rely on their working adult children for transportation purposes [21,73]. The same holds for items assessing the availability of free Wi-Fi and public housing for the elderly given that older immigrants are often among the most socially disadvantaged groups and financially dependent on their family [76,77]. Whilst items gauging the accessibility or prevalence of Chinese food outlets, information in Chinese, and Chinese-speaking residents, staff, officials, or formal and informal groups (e.g., clubs, volunteers) are obviously specific to older Chinese immigrants, they can be easily adapted for use with other ethnic groups of immigrants. An aspect of the physical food environment included in the NEHA-CIA that is particularly idiosyncratic to older Chinese immigrants is having food outlets with strict food and hygiene policies in the neighborhood [21]. Studies have reported that Chinese residents viewed food safety as one of the most significant health risks [78] because food safety has been and still remains a major concern in China [79]. Thus, it is not surprising that qualitative formative research underpinning the development of the NEHA-CIA identified this issue as an important aspect of the neighborhood physical food environment [21].

Items specifically relevant to older immigrants were also added to the Perceived Barriers to Health-Enhancing Behaviors questionnaire. These included items assessing language barriers to engaging in leisure-time physical activity and socializing, lack of information on activities, and health-related behaviors in Chinese and dependence on, and lack of support from, family members for engaging in specific health-enhancing behaviors. While the inclusion of language-related items does not necessitate any justification, the addition of items about family dynamics require some elaboration. As noted earlier, older immigrants are often socially disadvantaged and dependent on working members of their family (partner or children) [76,77]. This limits their mobility and freedom to participate in various physical and social activities in the community [21,73,74]. Furthermore, older immigrants’ disadvantaged socio-economic position and lower status in the household may restrict their ability to eat healthy foods because younger income-earning family members are more likely to adopt an unhealthy Western diet [80].

### 4.2. Metric Characteristics of Questionnaires

Substantial to excellent test-retest reliability was observed for virtually all subscales of the NEHA-CIA and Perceived Barriers to Health-Enhancing Behavior questionnaire. Additionally, adequate levels of internal consistency were found for most subscales with the exception of physical barriers to walking and social disorder/littering, which consisted of only two and three items. When compared to the interviewer-administered version of the NEWS-CS, the self-administered NEHA-CIA showed higher levels of reliability. For example, the ICCs for the residential density and street connectivity subscales of the NEHA-CIA were respectively 0.97 and 0.73 in the present study, while the same subscales of the NEWS-CS had ICCs of 0.72 and 0.58 [32]. These discrepancies may be due to the different administration modes [81], clearer meaning of the response options for the self-administered version, having the opportunity to spend more time filling out and memorizing the self-administered version of the questionnaire, and/or to differences in environmental settings. Prior studies comparing the test-retest reliability of self- and interviewer-administered versions of questionnaires have reported contrasting results [82,83]. However, these studies did not have different response scales across administration modes.

Of note is the fact that the test-retest reliability of the NEHA-CIA residential density subscale and its items was particularly high (ICC = 0.97). This may have been due the restricted variability of residential buildings in the areas of Melbourne (Australia) selected for this study. Most participants reported having only detached single-family and/or multi-family houses in their neighborhood and only two out of 52 participants reported having a few >6 storey buildings. While the test-retest reliability of the all subscales of both questionnaires was good, that of a handful of NEHA-CIA items was not acceptable. Two of these items pertained to the perceived time needed to walk from home to the nearest swimming pool and public toilet. Participants might have rarely visited or walked to these destinations from home, making them unsure about their location in space. In a validation study of the NEWS-CS, distance to the nearest public toilet was also one of the items with the lowest test-retest reliability (ICC = 0.29) [32]. The other two items with inadequate repeatability were “Shops in the neighborhood selling fresh fruit and vegetable juice” and “Parked cars block vision and make it difficult to cross the road”. Fresh fruit and vegetable juice may not be a particularly popular purchasing item among older Chinese immigrants and the number of parked cars on the road may substantially vary from day to day. The latter item showed only moderate test-retest reliability in the validation study of the NEWS-CS [32].

An interesting finding related to the NEHA-CIA was that participants tended to report greater perceived distances to several destinations (from home) in the second than the first assessment, which then also resulted in a significant between-assessment difference in average scores on the land use diversity subscale. Empirical evidence suggests that people tend to underestimate the time needed to reach a familiar destination via a familiar route [84,85]. It is possible that between the first and the second assessments, some participants verified the time needed to walk from home to specific familiar destinations included in the NEHA-CIA. This would have resulted in an increase in average estimated walking time from the first to the second assessment. Alternatively, these (few) differences might have occurred by chance as a substantial number of significance tests were performed.

With regard to the test-retest reliability of the Perceived Barriers to Health-Enhancing Behaviors questionnaire, only items of the leisure-time physical activity and walking for transport subscales were examined in previous studies. Pilot testing of an interviewer-administered version of these subscales in Hong Kong older adults resulted in ICCs ranging from 0.65 to 0.75 [61], which are in line with those observed in the present study. Previous studies also found levels of internal consistency for the perceived barriers to leisure-time physical activity subscale similar to this study, with Cerin and colleagues reporting a Cronbach’s α of 0.79 [86].

Apart from providing support for the repeatability of the newly-developed questionnaires, this study also provides support for their construct validity. In fact, most NEHA-CIA subscales measuring components of transport-related walkability (i.e., street connectivity, general access to services, and land use diversity) were positively related to an objectively-determined walkability index. Cerin and colleagues [31] reported similar findings for an Australian version of the NEWS administered to English-speaking adults. However, while the earlier study found a positive association between the residential density subscale and a walkability index, the present study did not. This was likely due to insufficient variability in residential building types in the study areas selected for the present study. It is also noteworthy that, similarly to the study on the Australian NEWS [31], we found positive associations between the walkability index and measures of access to public transport, pedestrian infrastructure, and traffic hazards. Pedestrian infrastructure and access to public transport are usually better, and traffic is usually heavier in destination-rich neighborhoods [31,50,87].

Further evidence of construct validity of the NEHA-CIA can be derived from a comparison of the average scores on the NEWS-CS subscales obtained from a sample of older Hong Kong dwellers [32] with those of the corresponding subscales of the NEHA-CIA found in the present sample of Melbournians. In 2016, the average population density in Hong Kong was 6760 people/km^2^ [88], while that in Greater Melbourne (Australia) was 472 people/km^2^ [89]. When compared to Melbourne, Hong Kong is a much denser metropolis with better access to a variety of services, transportation infrastructure, indoor places for walking, and with lower levels of crime [32,90,91,92]. Mirroring these differences between the two cities, Hong Kong older adults had significantly higher scores on several NEWS-CS/NEHA-CIA subscales than their Melbourne counterparts including residential density (678 vs. 51 on a scale from 6 to 789), land use diversity (3.9 vs. 2.5 on a 5-point scale), street connectivity (3.9 vs. 2.8 on a 4-point scale here and thereafter), general access to services (3.9 vs. 2.9), infrastructure for walking (3.9 vs. 2.7), and transportation (3.9 vs. 2.6). They also had lower average scores on the crime subscale (1.3 vs. 2.0) [32].

Construct validity for the NEHA-CIA subscales measuring aspects of the neighborhood food and social environments was supported by the positive associations between these subscales and the percentage of Chinese residents in the neighborhood. It is plausible to expect that communities with a higher percentage of Chinese residents would have better access to Chinese ethnic foods, media, and activities in Chinese, and Chinese-speaking staff.

In this study, construct validity of the Perceived Barriers to Health-Enhancing Behaviors questionnaire was examined by estimating the associations between the various subscales and objective and/or perceived environmental attributes that might be theoretically related to behavior-specific barriers. Only a few weak associations in the expected directions were observed between perceived barriers to a specific behavior and perceived social and physical aspects of the neighborhood environment associated with the same behavior. For example, perceived access to recreational facilities was negatively related to perceived barriers to leisure-time physical activity, a finding supported by previous studies [86]. The general lack of associations could be attributed to the fact that the perceived barriers subscales included many items capturing individual-level personal factors such as lack of motivation, time, enjoyment, and health unrelated to environmental factors. A better test of the construct validity of the perceived barriers subscales would involve an examination of their relationships with the behavior of reference.

### 4.3. Limitations and Future Research

This study has several limitations. It was based on a relatively small purposive sample of participants recruited from a limited number of demographically and physically diverse but prevalently medium-income neighborhoods in metropolitan Melbourne. A larger sample of participants from a larger number of socio-economically diverse neighborhoods might have resulted in greater variability in responses and, hence, more power to test the construct validity of the various subscales. It would have also enabled us to test the factorial validity of the two questionnaires. Nevertheless, the current sample was sufficiently large to examine the repeatability and internal consistency of the subscales [93], which was the primary aim of the study.

This study did not collect information on individual-level socio-economic indicators such as educational attainment and household income, which might have provided further insight into the validity and appropriateness of the questionnaires. Data on household income were not collected because older Chinese immigrants are usually dependent on their adult children [21] and our pilot work indicated that they might be unlikely to know or be willing to report such information. Furthermore, since our study developed self-administered questionnaires that required participants to be able to read and write in Chinese, we did not collect information on their educational attainment and assumed that they would have completed either primary or secondary education.

The fact that the transportation subscale of the NEHA-CIA had a relatively low level of internal consistency despite consisting of a substantial number of items indicates that it may not be unidimensional. Future studies would need to examine the factorial structure of this as well as other newly-developed subscales (e.g., destinations for socializing and physical food environment) not included in previous versions of the NEWS. Future studies will need to examine the metric characteristics of the NEHA-CIA and perceived barriers questionnaire on a more representative sample of older Chinese immigrants residing in various Australian cities. They also need to further examine the construct validity by estimating the associations with the target health-enhancing behaviors (physical activity, healthy eating, and socializing) and identify potential floor and ceiling effects. As noted earlier, both questionnaires could also be adapted to other ethnic groups of older immigrants to Australia and other Western developed countries.

The questionnaires we developed in this study contain a large number of items that are relevant to adults and older adults in general, irrespective of their ethnicity, and a smaller number of items that are specifically pertinent to Chinese older adults. This makes it possible for future research employing relevant sections of the NEHA-CIA and Perceived Barriers to Health-Enhancing Behaviors questionnaire to examine and compare perceived neighborhood attributes and barriers to engagement in health-enhancing behaviors across older Chinese immigrants, other ethnic groups, and mainstream older urban dwellers in Australia. It also makes it possible to study between-group differences and similarities in the explanatory power of perceived environmental attributes and barriers in relation to the actual health-enhancing behaviors (physical activity, healthy eating, and socializing). This information is important for the development of environmental and public health interventions that equitably benefit various segments of the population.

## 5. Conclusions

We developed/adapted and preliminarily validated questionnaires appropriate for older Chinese immigrants to Australia measuring perceived aspects of the neighborhood environment affecting physical activity, healthy eating, and socializing (the NEHA-CIA), and perceived barriers to the same health-enhancing behaviors. The questionnaires capture issues applicable to older adults in general as well as issues specifically pertinent to older Chinese immigrants and can be potentially adapted to other ethnic groups of immigrants. Most of the subscales of the two questionnaires showed good test-retest reliability and internal consistency, and the NEHA-CIA also displayed good construct validity. Future research needs to further examine the construct validity of the perceived barriers to health-enhancing behaviors questionnaire and test the factorial validity of both measures in a representative sample of older Chinese immigrants.

## Figures and Tables

**Table 1:** Participant socio-demographic and health-related characteristics by study stage.

		Cognitive Interviews (*n* = 31)		Quantitative Stage (*n* = 52)	
		*n*	%	*n*	%
Neighborhood walkability	High	18	58.1	22	42.3
	Low	13	41.9	30	57.7
Neighborhood % Chinese	Medium	11	35.5	24	46.2
	High	20	64.5	28	53.8
Sex	Male	13	41.9	20	38.5
	Female	18	58.1	32	61.5
Age group	60–64 years	4	12.9	8	15.4
	65–69 years	5	16.1	14	26.9
	70–74 years	6	19.4	15	28.8
	75–79 years	11	35.5	12	23.1
	80+ years	5	16.1	3	5.8
Place of origin	Shanghai municipality	7	22.6	15	28.8
	Guangdong Province	1	3.2	12	23.1
	Beijing municipality	7	22.6	3	5.8
	Other areas ^a^	10	32.3	14	26.9
	Hong Kong SAR	5	16.1	6	11.5
	Taiwan	1	3.2	0	0.0
	Other ^b^	0	0.0	2	3.9
Language spoken at home	Mandarin only	25	80.6	32	61.5
	Cantonese only	5	16.1	18	34.6
	Other ^c^	1	3.2	2	3.9
Residency in Australia	<5 years	11	35.5	25	48.1
	5–14 years	9	29.0	9	17.3
	15–24 years	5	16.1	10	19.2
	25+ years	6	19.4	8	15.4
Living arrangements	Living alone	3	9.7	4	7.7
	Living with others but not adult children	12	38.7	22	42.3
	Living with adult children	16	51.6	26	50.0
Self-rated health	Poor	1	3.2	4	7.7
	Fair	16	51.6	19	36.5
	Good	7	22.6	21	40.4
	Very good	7	22.6	7	13.5
	Excellent	0	0.0	1	1.9
Number of chronic health conditions	None	9	29.0	20	38.5
	One	7	22.6	18	34.6
	Two or more	15	48.4	14	26.9

^a^ Other includes Chongqing, Sichuang, Anhui, Fujian, Jiangsu, Shandong, Zhejiang, Heilongjiang, Liaoning, Shanxi, Tianjin provinces/municipalities. ^b^ Other includes Malaysia, Vietnam. ^c^ Other includes English, Chiu Chau dialect, Shang Hai dialect, Southern Fujian dialect, Vietnamese, Taiwanese or any combination of these with Mandarin and/or Cantonese.

**Table 2:** Initial questionnaire items of perceived neighborhood attributes associated with physical activity, healthy eating, and socializing appropriate for older Chinese immigrants to Australia: content, sources of items, and modifications prior to administration to participants (Neighborhood Environment for Healthy Aging–Chinese Immigrants to Australia).

Subscales/Items	Source	Behavior Affected	Modifications to Subscale Prior to Administration to Participants in Cognitive Interviews/Notes
General instructions	NEWS-CS	N/A	None
***Residential density*** [6 items]	NEWS-CS	PA, HE, S	None
***Land use diversity*** (distance to destinations) [30 items]	NEWS-CS	PA, HE, S	Replaced item “*Hong Kong Jockey Club betting branch*” with “*Lottery/betting outlet*”.
New item [1 item]: - *Distance of ‘own garden/vegetable patch’ from home*	NGT sessions	PA, HE	Added item to Land use diversity subscale. “*Own garden/vegetable patch*” was identified as a destination for physical activity and a source of healthy foods.
***General access to services*** [3 items]	NEWS-CS	PA, S	None
***Recreational facilities*** [4 items] - *The recreational facilities in my neighborhood are affordable* - *There are many recreational facilities in my neighborhood* - *Recreational centers in my neighborhood provide a variety of activities for the elderly* - *Recreational centers in my neighborhood have Chinese-speaking instructors and volunteers*	NGT sessions	PA, S	New subscale developed based on NGT session findings suggesting that the number and affordability of recreational facilities, and the suitability of activities for Chinese elderly influence the likelihood of participation in physical activity. As access to destinations that support group activities have been also reported to facilitate social engagement, this subscale is also deemed to be relevant to socializing.
***Physical food environment*** [2 items]	MESA FE items	HE	New subscale. Two items taken from the MESA food environment scale without modifications.
New items [8 items]: - *There are many grocery shops in my neighborhood* - *Food outlets in our local area implement strict food and hygiene policies* - *Healthy foods cost too much* - *Some food shops in my neighborhood have an organic food section* - *Foods sold in shops have Chinese food labelling indicating whether the product is a healthy option* - *There is an Asian grocery shop nearby* - *Residents in my neighborhood can grow vegetables in their own garden* - *Some shops in my neighborhood sell fresh fruit and vegetable juices*	NGT sessions	HE	Eight new items added to the physical food environment subscale based on findings from NGT sessions indicating that having many grocery stores in the neighborhood that implement strict hygiene practices and sell affordable Asian fresh organic foods with Chinese food labelling would promote healthy eating in older Chinese immigrants. Having the space and ability to grow one’s own vegetables was also considered an important facilitator of healthy eating.
***Destinations for socializing*** [5 items] - *There is a Chinese senior club in my neighborhood* - *Our neighborhood provides public housing for the elderly* - *My neighborhood has many destinations and services where people can meet* - *There is a Chinese library in my neighborhood providing a convenient meeting point* - *There is a free Wi-fi in my neighborhood that makes it possible for me to keep in touch with my friends and family*	NGT sessions	S	New subscale developed based on findings from the NGT sessions identifying places and facilities in the neighborhood that would facilitate social interaction in older Chinese immigrants to Australia.
***Transportation*** [1 item]	NEWS-CS	PA, S	New subscale. One item taken from NEWS-CS without modifications.
New items [7 items]: - *Parking is too expensive* - *Public transport is easy to use* - *Buses, trains and trams provide clear information on the next stop (stop name or number) so that I know when I need to get off.* - *Public transport in my area is infrequent* - *Public transport services are too expensive* - *Transit stop and public transport signage is poor (e.g., cannot read transit stop number or bus number)* - *There is no convenient and affordable public transport to get to/from grocery shops*	NGT sessions	PA, HE, S	Seven new items added to the transportation subscale. NGT sessions identified public transport availability, user-friendliness and frequency as important factors impacting on the ability to participate in physical and social activities. Affordable and convenient public transport to/from grocery shops emerged as a facilitator of healthy eating.
***Physical barriers to walking*** [3 items]	NEWS-CS	PA, S	None
***Street connectivity*** [2 items]	NEWS-CS	PA	None
***Infrastructure for pedestrians*** [7 items]	NEWS-CS	PA, S	Merged the infrastructure for walking subscale of the NEWS-CS with four related items including: sitting facilities; easy access of residential entrance; bridge/overpass connecting to services; fence or grass strip separating the footpath and traffic.
***Indoor places for walking*** [2 items]	NEWS-CS	PA, S	None
***Aesthetics*** [4 items]	NEWS-CS	PA	None
***Traffic and road hazards*** [6 items]	NEWS-CS	PA	None
***Traffic speed*** [2 items]	NEWS-CS	PA	None
***Social disorder/littering*** [2 items]	NEWS-CS	PA, S	None
***Crime*** [3 items]	NEWS-CS	PA, S	None
***Safety—presence of people*** [2 items]	NEWS-CS	PA, S	None
***Social environment and communication–physical activity*** [2 items] - *Our local volunteer organizations organize outdoor physical activity sessions* - *I can easily find information in Chinese on health, community policies and community services promoting physical activity in my neighborhood*	NGT sessions	PA, S	New subscale developed based on findings from the NGT sessions identifying social activities and communication channels that facilitate engagement in physical activity in older Chinese immigrants to Australia.
***Social environment and communication–healthy eating*** [3 items] - *There are people in my neighborhood who I can talk to about healthy eating* - *Our local Chinese newspaper/magazine publishes information on healthy eating* - *Our local community organizes talks on healthy eating in Chinese*	NGT sessions	HE, S	New subscale developed based on findings from the NGT sessions identifying social activities and dissemination of information in the neighborhood that promote healthy eating in older Chinese immigrants to Australia.
***Social environment and communication–socializing*** [5 items] - *Volunteers organize free-of-charge group activities for Chinese elders in our neighborhood* - *Clinics, shops and other services in my neighborhood have staff who can speak English and Chinese* - *Our neighborhood provides formal or informal opportunities for Chinese- and English-speaking older residents to network* - *There are many Chinese-speaking residents in my neighborhood* - *There are Chinese-speaking policemen in my neighborhood*	NGT sessions	S	New subscale developed based on findings from the NGT sessions identifying neighborhood demo- graphics and social activities in the neighborhood that promote socializing in older Chinese immigrants to Australia.

*Notes.* NGT = nominal group technique; N/A = not applicable; PA = physical activity; HE = healthy eating; S = socializing; NEWS-CS = Neighborhood Environment Walkability Scales-Chinese Seniors; MESA FE = Multi-Ethnic Study of Atherosclerosis Food Environment.

**Table 3:** Initial questionnaire items of perceived barriers to engagement in, physical activity, healthy eating, and socializing appropriate for older Chinese immigrants to Australia: content, sources of items, and modifications prior to administration to participants (Perceived barriers to Engaging in Health-Enhancing Behaviors questionnaire).

Subscales/Items	Source	Behavior Affected	Modifications to Subscale Prior to Administration to Participants in Cognitive Interviews/Notes
***Perceived barriers to leisure-time physical activity*** [14 items]	Perceived barriers to PA NGT sessions	PA	Merged items “*lack of skills*” and “*lack of knowledge*”. Modified “*Lack of facilities, space or places to walk*” to “*Lack of facilities, community activities for Chinese, space or places to walk*” based on findings from NGT sessions.
Item [1 item] - *Air pollution*	ALECS study	PA	Added *“air pollution”* as a barrier to engaging in outdoor physical activity.
New items [6 items]: - *Lack of money* - *Inability to communicate with others in English* - *Lack of information on community activities* - *Difficulties in getting to/from recreational destination (e.g., infrequent public transport)* - *Being dependent on other family members and lacking freedom*	NGT sessions	PA	Six new items added to the scale of perceived barriers to leisure-time physical activity. NGT sessions identified language barriers, lack of information on physical activity opportunities, financial dependence on the family and transportation difficulties.
***Perceived barriers to walking for transport*** [8 items]	Perceived barriers to PA	PA	Modified “*Lack of facilities, space or places to walk*” to *“Lack of places to walk to”*.
Items [4 items]: - *Air pollution* - *Physical barriers (e.g., steep hills) to destinations* - *Traffic hazards* - *Crime*	ALECS study	PA	Added environmental barriers to engaging in walking for transport that were included in studies on Chinese older adults.
***Perceived barriers to healthy eating*** [9 items] - *Not enjoying eating healthy foods* - *Healthy foods being too expensive* - *Lack of time to prepare healthy foods* - *Lack of energy/motivation to prepare healthy foods* - *Lack of skills to plan, shop for, prepare or cook healthy foods* - *Lack of healthy options in food shops* - *Lack of friends’ support for healthy eating* - *Lack of partner’s support for healthy eating* - *Lack of children’s support for healthy eating*	Pan-EU survey NGT sessions	HE	Provided more detailed description of factors included in a list of barriers to healthy eating used in the Pan-EU survey (unappealing foods; price; busy lifestyle; lengthy preparation; willpower; cooking skills; healthy options unavailable; taste preferences of family and friends) in line with findings from the NGT sessions.
New items [3 items]: - *Lack of information about a healthy diet in Chinese* - *Grocery stores are too far away from home* - *Eating out*	NGT sessions	HE	Three additional items were included in the scale of Perceived barriers to healthy eating. NGT sessions identified language barriers, transportation difficulties and eating out as frequent barriers to healthy eating among older Chinese immigrants to Australia.
***Perceived barriers to socializing*** [5 items] - *Lack of good health* - *Shyness/introversion* - *Lack of time* - *Lack of motivation* - *Poor public transport*	Various studies NGT sessions	S	Factors included based on findings from studies investigating barriers to social engagement in older adults and refined based on findings from NGT sessions. Poor health, lack of motivation, lack of time, transportation problems and shyness/introversion have been often identified as important factors impacting on social participation.
New items [6 items]: - *Inability to speak English* - *Lack of activity centers and facilities for Chinese elderly* - *Lack of Chinese-speaking people* - *Lack of information on events, meeting groups for Chinese elderly* - *Lack of partner’s support for socializing* - *Lack of children’s support for socializing*	NGT sessions	S	Six additional items were included in the scale of perceived barriers to socializing. NGT sessions identified several barriers related to language and culture. Lack of support for socializing from family members was also identified as a barrier to socializing among older Chinese immigrants to Australia.

*Notes.* NGT = nominal group technique; PA = physical activity; HE = healthy eating; S = socializing; ALECS = Active Lifestyle and the Environment in Chinese Seniors.

**Table 4:** Summary of modifications to questionnaires of perceived neighborhood attributes associated with, and perceived barriers to engagement in, physical activity, healthy eating, and socializing for older Chinese immigrants to Australia based on findings from cognitive interviews.

**Questionnaire: Neighborhood Environment for Healthy Aging-Chinese Immigrants to Australia**
Added instructions to front page: “*Please remember that there are no right or wrong answers*”; “*Please keep in mind that the questions are about Australia and the current situation in your neighborhood*”. Added instructions to each subscale: “*Please put a check mark “√” next to your answer (Please only use one check mark “√” for each question).”* Added instructions to all subscales except for Land use diversity”: *“Neighborhood or ‘easy to walk’ means within 1 km or 10–15 min walking distance from where you live.”* *“The statements below are about Australia and the current situation in your neighborhood.”* *“Agree-means that you agree that the statement is true.”* *“Disagree-means that you do not think that the statement is true.”* When appropriate, clarified in each item that “neighborhood” means *“within 1 km or 10–15 min walking distance”.* Replaced rating scale anchors *“somewhat agree”* and *“somewhat disagree”* with *“agree”* and *“disagree”*. Added the response option *“unsure”* between *“disagree”* and *“agree”*. Provided *“Not applicable”* as a response option, when appropriate. Provided a definition/explanation/example/picture for words whose meaning was unclear to participants (e.g., healthy diet; hardware store; Western non-fast-food restaurant). Reworded items to improve clarity (e.g., changed ‘our local Chinese newspaper” to “our Australian local Chinese newspaper’). Added items to scales (e.g., two-dollar shop in the land use diversity subscale; subitems for distinct public transport modes [bus, train, and tram] in the transportation subscale).
**Questionnaire: Perceived Barriers to Engaging in Health-Enhancing Behaviors**
Provided an explanation of the health- enhancing behavior examined in each section (e.g., “*This section is about regular exercise, including walking for recreational purposes (e.g., walking in leisure time, Tai-Chi, etc.*).” Changed instructions and item structure from “*How often do the following prevent you from [engaging in a specific health-enhancing behavior]? (Please circle the most appropriate answer–one response per question)*”. [Specific barrier] Never Rarely Sometimes Often Very often 1 2 3 4 5 to: “*Do the following reasons prevent you from [engaging in a specific health-enhancing behavior]?* *Please circle the most appropriate answer–one response per question.”* Example: “[Specific barrier] [often] prevents me from [engaging in a specific health-enhancing behavior.” Never Rarely Sometimes Often Very often 1 2 3 4 5 Provided a definition/explanation/example for words whose meaning was unclear to participants (e.g., self-conscious about my looks; lack of self-discipline; traffic hazards). Provided *“Not applicable”* as a response option, when appropriate.

**Table 5:** Descriptive statistics, test-retest reliability, and internal consistency of measures of perceived neighborhood environmental attributes influencing, and perceived barriers to engagement in, healthy behaviors for older Chinese immigrants to Australia.

Measure [Theoretical Range; Number of Items]	*M* (SD) [Assessment 1]	*M* (SD) [Assessment 2]	ICC (95% CI)	Cronbach α
*Questionnaire: Neighborhood Environment for Healthy Aging–Chinese Immigrants to Australia*				
Residential density [6–789; 6 items]	51.06 (8.93)	52.98 (9.58)	0.97 (0.94, 0.98)	N/A
Land use diversity (proximity of destinations) ^#^ [1–5; 31 items]	2.48 (0.62)	2.53 (0.62) *	0.77 (0.65, 0.87)	N/A
General access to services ^§^ [1–4; 3 items]	2.91 (0.52)	2.89 (0.47)	0.73 (0.58, 0.84)	0.87
Recreational facilities ^§^ [1–4; 4 items]	2.57 (0.87)	2.55 (0.83)	0.79 (0.65, 0.87)	0.72
Physical food environment ^§^ [1–4; 10 items]	2.57 (0.48)	2.58 (0.48)	0.80 (0.62, 0.89)	0.88
Destinations for socializing ^§^ [1–4; 5 items]	2.35 (0.58)	2.35 (0.62)	0.95 (0.91, 0.97)	0.78
Transportation ^§^ [1–4; 18 items]	2.61 (0.24)	2.62 (0.24)	0.78 (0.61, 0.87)	0.68
Physical barriers to walking ^§^ [1–4; 3 items]	2.35 (0.41)	2.29 (0.39) *	0.89 (0.82, 0.94)	0.40
Street connectivity ^§^ [1–4; 2 items]	2.81 (0.44)	2.82 (0.41)	0.73 (0.56, 0.83)	0.81
Infrastructure for pedestrians ^§^ [1–4; 7 items]	2.66 (0.35)	2.63 (0.29)	0.75 (0.56, 0.85)	0.71
Indoor places for walking ^§^ [1–4; 2 items]	2.35 (0.61)	2.31 (0.68)	0.77 (0.61, 0.87)	0.84
Aesthetics ^§^ [1–4; 4 items]	2.66 (0.41)	2.68 (0.48)	0.83 (0.72, 0.90)	0.75
Traffic and road hazards ^§^ [1–4; 6 items]	1.97 (0.28)	2.03 (0.30) *	0.79 (0.65, 0.88)	0.79
Traffic speed ^§^ [1–4; 2 items]	2.76 (0.50)	2.75 (0.51)	0.67 (0.28, 0.91)	0.67
Social disorder/littering ^§^ [1–4; 2 items]	1.88 (0.37)	1.90 (0.24)	0.68 (0.50, 0.80)	0.44
Crime ^§^ [1–4; 3 items]	1.98 (0.49)	2.04 (0.42)	0.77 (0.63, 0.86)	0.77
Safety—Presence of people ^§^ [1–4; 2 items]	2.75 (0.53)	2.69 (0.53)	0.89 (0.81, 0.93)	0.80
Social environment and communication—Physical activity ^§^ [1–4; 2 items]	2.14 (0.60)	2.16 (0.65)	0.77 (0.68, 0.82)	0.73
Social environment and communication—Healthy eating ^§^ [1–4; 3 items]	2.51 (0.54)	2.54 (0.43)	0.59 (0.10, 0.81)	0.64
Social environment and communication—Socializing ^§^ [1–4; 5 items]	2.36 (0.59)	2.33 (0.57)	0.64 (0.48, 0.74)	0.84
*Questionnaire: Perceived barriers to engaging in health-enhancing behaviors*				
Perceived barriers to leisure-time physical activity [1–5; 20 items]	2.10 (0.48)	2.09 (0.46)	0.73 (0.53, 0.84)	0.86
Perceived barriers to walking for transport [1–5; 12 items]	1.95 (0.43)	1.94 (0.41)	0.73 (0.58, 0.84)	0.75
Perceived barriers to healthy eating [1–5; 12 items]	2.29 (0.67)	2.29 (0.70)	0.77 (0.61, 0.85)	0.84
Perceived barriers to socializing [1–5; 11 items]	2.45 (0.52)	2.46 (0.55)	0.70 (0.64, 0.74)	0.72

*Notes.*^#^ Original scale reverse coded to represent proximity rather than distance. ^§^ Rescaled from a 5-point to a 4-point scale to allow comparison with previous studies. * *p* < 0.05 (difference between means).

**Table 6:** Associations of measures of perceived neighborhood environmental attributes influencing, and perceived barriers to engagement in, healthy behaviors for older Chinese immigrants to Australia with theoretically-related constructs: construct validity analyses.

Measure [Theoretical Range]	Theoretically-Related Construct	*b*	ICC (95% CI)	*p*-Value
*Questionnaire: Neighborhood Environment for Healthy Aging–Chinese Immigrants to Australia*				
Residential density [6–789]	Walkability (ref: low)	−3.35	−64.26, 57.57	0.910
Land use diversity (proximity of destinations) ^#^ [1–5]	Walkability (ref: low)	0.42	0.09, 0.75	0.013
General access to services ^§^ [1–4]	Walkability (ref: low)	0.35	0.09, 0.62	0.010
Recreational facilities ^§^ [1–4]	Walkability (ref: low)	−0.02	−0.55, 0.50	0.926
Physical food environment ^§^ [1–4]	Walkability (ref: low)	0.26	−0.03, 0.54	0.073
	Percentage Chinese residents (ref: medium)	0.33	0.06, 0.59	0.018
Destinations for socializing ^§^ [1–4]	Walkability (ref: low)	0.09	−0.19, 0.37	0.513
	Percentage Chinese residents (ref: medium)	0.51	0.28, 0.74	<0.001
Transportation ^§^ [1–4]	Walkability (ref: low)	0.18	0.05, 0.32	0.009
Physical barriers to walking ^§^ [1–4]	Walkability (ref: low)	−0.05	−0.30, 0.19	0.670
Street connectivity ^§^ [1–4]	Walkability (ref: low)	0.02	0.003, 0.46	0.047
Infrastructure for pedestrians ^§^ [1–4]	Walkability (ref: low)	0.25	0.09, 0.41	0.003
Indoor places for walking ^§^ [1–4]	Walkability (ref: low)	0.31	−0.05, 0.66	0.086
Aesthetics ^§^ [1–4]	Walkability (ref: low)	0.07	−0.18, 0.32	0.601
Traffic and road hazards ^§^ [1–4]	Walkability (ref: low)	0.18	0.01, 0.35	0.040
Traffic speed ^§^ [1–4]	Walkability (ref: low)	0.21	−0.09, 0.51	0.164
Social disorder/littering ^§^ [1–4]	Walkability (ref: low)	0.05	−0.17, 0.27	0.633
Crime ^§^ [1–4]	Walkability (ref: low)	0.12	−0.13, 0.37	0.333
Safety—Presence of people ^§^ [1–4]	Walkability (ref: low)	0.34	0.04, 0.63	0.026
	Percentage Chinese residents (ref: medium)	0.41	0.12, 0.69	0.006
Social environment and communication—Physical activity ^§^ [1–4]	Walkability (ref: low)	0.19	−0.15, 0.53	0.262
	Percentage Chinese residents (ref: medium)	0.28	−0.03, 0.60	0.075
Social environment and communication—Healthy eating ^§^ [1–4]	Walkability (ref: low)	0.22	−0.03, 0.47	0.077
	Percentage Chinese residents (ref: medium)	0.27	0.04, 0.51	0.025
Social environment and communication—Socializing ^§^ [1–4]	Walkability (ref: low)	0.20	−0.14, 0.53	0.243
	Percentage Chinese residents (ref: medium)	0.77	0.50, 1.03	<0.001
*Questionnaire: Perceived barriers to engaging in health-enhancing behaviors*				
Perceived barriers to leisure-time physical activity [1–5]	Walkability (ref: low)	0.12	−0.15, 0.39	0.380
	Percentage Chinese residents (ref: medium)	−0.20	−0.47, 0.06	0.135
	Recreational facilities [1–4]	−0.24	−0.38, −0.09	0.002
	Social environment and communication–Physical activity [1–4]	−0.11	−0.32, 0.09	0.272
	Aesthetics [1–4]	−0.15	−0.43, 0.12	0.269
Perceived barriers to walking for transport [1–5]	Walkability (ref: low)	0.16	−0.09, 0.42	0.202
	Percentage Chinese residents (ref: medium)	0.10	−0.14, 0.34	0.402
	Residential density [6–789]	−0.001	−0.003, 0.001	0.373
	Land use diversity (proximity to destinations) [1–5]	−0.08	−0.27, 0.11	0.398
	General access to services [1–4]	−0.05	−0.30, 0.20	0.685
	Recreational facilities [1–4]	−0.11	−0.24, 0.03	0.121
	Physical food environment [1–4]	−0.06	−0.30, 0.19	0.640
	Destinations for socializing [1–4]	−0.15	−0.39, 0.08	0.186
	Transportation [1–4]	−0.08	−0.56, 0.41	0.747
	Physical barriers to walking [1–4]	0.02	−0.28, 0.33	0.885
	Traffic and road hazards [1–4]	−0.01	−0.42, 0.40	0.965
	Crime [1–4]	0.13	−0.14, 0.41	0.318
Perceived barriers to healthy eating [1–5]	Walkability (ref: low)	−0.13	−0.53, 0.26	0.502
	Percentage Chinese residents (ref: medium)	−0.18	−0.57, 0.21	0.355
	Physical food environment [1–4]	−0.35	−0.76, 0.05	0.087
	Social environment and communication–Healthy eating [1–4]	−0.04	−0.51, 0.43	0.872
	Transportation [1–4]	−0.33	−1.14, 0.48	0.423
Perceived barriers to socializing [1–5]	Walkability (ref: low)	−0.15	−0.45, 0.16	0.341
	Percentage Chinese residents (ref: medium)	−0.11	−0.41, 0.19	0.463
	Destinations for socializing [1–4]	−0.11	−0.51, 0.21	0.503
	Social environment and communication—Socializing [1–4]	−0.25	−0.51, 0.02	0.068

*Notes.*^#^ Original scale reverse coded to represent proximity rather than distance. ^§^ Rescaled from a 5-point to a 4-point scale to allow comparison with previous studies. ref = Reference category.

## Data Availability

The data supporting the reported results can be found by contacting the corresponding author.

## Supplementary Materials

The following are available online at https://www.mdpi.com/article/10.3390/ijerph18094531/s1, Table S1: Questionnaires of perceived neighborhood attributes associated with, and perceived barriers to engagement in, physical activity, healthy eating, and socializing appropriate for older Chinese immigrants to Australia: detailed description of modifications based on findings from cognitive interviews; Table S2: Descriptive statistics, test-retest reliability and internal consistency of measures of perceived neighborhood environmental attributes influencing, and perceived barriers to engagement in, healthy behaviors for older Chinese immigrants to Australia; Final versions of Neighborhood Environment for Healthy Aging–Chinese Immigrants to Australia and Perceived Barriers to Health-Enhancing Behaviors questionnaires.

## References

[1] Bellavia A., Bottai M., Wolk A., Orsini N. (2013). Physical activity and mortality in a prospective cohort of middle-aged and elderly men—A time perspective. Int. J. Behav. Nutr. Phys. Act..

[2] Sofi F., Cesari F., Abbate R., Gensini G.F., Casini A. (2008). Adherence to Mediterranean diet and health status: Meta-analysis. BMJ.

[3] McCullough M.L., Patel A.V., Kushi L.H., Patel R., Willett W.C., Doyle C., Thun M.J., Gapstur S.M. (2011). Following cancer prevention guidelines reduces risk of cancer, cardiovascular disease, and all-cause mortality. Cancer Epidemiol. Biomark. Prev..

[4] Myers J., McAuley P., Lavie C.J., Despres J.P., Arena R., Kokkinos P. (2015). Physical activity and cardiorespiratory fitness as major markers of cardiovascular risk: Their independent and interwoven importance to health status. Prog. Cardiovasc. Dis..

[5] Babio N., Bullo M., Salas-Salvado J. (2009). Mediterranean diet and metabolic syndrome: The evidence. Public Health Nutr..

[6] Colberg S.R., Sigal R.J., Fernhall B., Regensteiner J.G., Blissmer B.J., Rubin R.R., Chasan-Taber L., Albright A.L., Braun B., American College of Sports M. (2010). Exercise and type 2 diabetes: The American College of Sports Medicine and the American Diabetes Association: Joint position statement. Diabetes Care.

[7] Livingston G., Huntley J., Sommerlad A., Ames D., Ballard C., Banerjee S., Brayne C., Burns A., Cohen-Mansfield J., Cooper C. (2020). Dementia prevention, intervention, and care: 2020 report of the Lancet Commission. Lancet.

[8] Kawachi I., Colditz G.A., Ascherio A., Rimm E.B., Giovannucci E., Stampfer M.J., Willett W.C. (1996). A prospective study of social networks in relation to total mortality and cardiovascular disease in men in the USA. J. Epidemiol. Community Health.

[9] Cacioppo J.T., Hawkley L.C., Berntson G.G., Ernst J.M., Gibbs A.C., Stickgold R., Hobson J.A. (2002). Do lonely days invade the nights? Potential social modulation of sleep efficiency. Psychol. Sci..

[10] Welin L., Tibblin G., Svardsudd K., Tibblin B., Ander-Peciva S., Larsson B., Wilhelmsen L. (1985). Prospective study of social influences on mortality. The study of men born in 1913 and 1923. Lancet.

[11] Stokols D. (1992). Establishing and maintaining healthy environments. Toward a social ecology of health promotion. Am. Psychol..

[12] Cerin E., Nathan A., van Cauwenberg J., Barnett D.W., Barnett A., Council on Environment and Physical Activity—Older Adults Working Group (2017). The neighbourhood physical environment and active travel in older adults: A systematic review and meta-analysis. Int. J. Behav. Nutr. Phys. Act..

[13] Van Cauwenberg J., Nathan A., Barnett A., Barnett D.W., Cerin E., Council on Environment and Physical Activity—Older Adults Working Group (2018). Relationships between Neighbourhood Physical Environmental Attributes and Older Adults’ Leisure-Time Physical Activity: A Systematic Review and Meta-Analysis. Sports Med..

[14] Sharkey J.R., Johnson C.M., Dean W.R. (2010). Food access and perceptions of the community and household food environment as correlates of fruit and vegetable intake among rural seniors. BMC Geriatr..

[15] Schmidt T., Kerr J., Schipperijn J. (2019). Associations between Neighborhood Open Space Features and Walking and Social Interaction in Older Adults-A Mixed Methods Study. Geriatrics.

[16] Barnett A., Zhang C.J.P., Johnston J.M., Cerin E. (2018). Relationships between the neighborhood environment and depression in older adults: A systematic review and meta-analysis. Int. Psychogeriatr..

[17] Rachele J.N., Sugiyama T., Davies S., Loh V.H.Y., Turrell G., Carver A., Cerin E. (2019). Neighbourhood built environment and physical function among mid-to-older aged adults: A systematic review. Health Place.

[18] Besser L.M., McDonald N.C., Song Y., Kukull W.A., Rodriguez D.A. (2017). Neighborhood Environment and Cognition in Older Adults: A Systematic Review. Am. J. Prev. Med..

[19] Cerin E., Rainey-Smith S.R., Ames D., Lautenschlager N.T., Macaulay S.L., Fowler C., Robertson J.S., Rowe C.C., Maruff P., Martins R.N. (2017). Associations of neighborhood environment with brain imaging outcomes in the Australian Imaging, Biomarkers and Lifestyle cohort. Alzheimers Dement..

[20] Lin X., Bryant C., Boldero J., Dow B. (2016). Psychological well-being of older Chinese immigrants living in Australia: A comparison with older Caucasians. Int. Psychogeriatr..

[21] Cerin E., Nathan A., Choi W.K., Ngan W., Yin S., Thornton L., Barnett A. (2019). Built and social environmental factors influencing healthy behaviours in older Chinese immigrants to Australia: A qualitative study. Int. J. Behav. Nutr. Phys. Act..

[22] United Nations Department of Economic and Social Affairs (2019). International Migrant Stock 2019.

[23] Hoehner C.M., Brennan Ramirez L.K., Elliott M.B., Handy S.L., Brownson R.C. (2005). Perceived and objective environmental measures and physical activity among urban adults. Am. J. Prev. Med..

[24] Cerin E., Conway T.L., Adams M.A., Barnett A., Cain K.L., Owen N., Christiansen L.B., van Dyck D., Mitas J., Sarmiento O.L. (2018). Objectively-assessed neighbourhood destination accessibility and physical activity in adults from 10 countries: An analysis of moderators and perceptions as mediators. Soc. Sci. Med..

[25] Cerin E., O’Connor T.M., Mendoza J.A., Thompson D., Lee R.E., Hughes S., Baranowski T. (2015). A child-centered scale of informal social control for Latino parents of preschool-aged children: Development and validation. Hisp. J. Behav. Sci..

[26] Echeverria S.E., Diez-Roux A.V., Link B.G. (2004). Reliability of self-reported neighborhood characteristics. J. Urban. Health.

[27] Cerin E., Conway T.L., Cain K.L., Kerr J., De Bourdeaudhuij I., Owen N., Reis R.S., Sarmiento O.L., Hinckson E.A., Salvo D. (2013). Sharing good NEWS across the world: Developing comparable scores across 12 countries for the Neighborhood Environment Walkability Scale (NEWS). BMC Public Health.

[28] Spittaels H., Foster C., Oppert J.M., Rutter H., Oja P., Sjostrom M., De Bourdeaudhuij I. (2009). Assessment of environmental correlates of physical activity: Development of a European questionnaire. Int. J. Behav. Nutr. Phys. Act..

[29] Oyeyemi A.L., Kasoma S.S., Onywera V.O., Assah F., Adedoyin R.A., Conway T.L., Moss S.J., Ocansey R., Kolbe-Alexander T.L., Akinroye K.K. (2016). NEWS for Africa: Adaptation and reliability of a built environment questionnaire for physical activity in seven African countries. Int. J. Behav. Nutr. Phys. Act..

[30] Nyunt M.S., Shuvo F.K., Eng J.Y., Yap K.B., Scherer S., Hee L.M., Chan S.P., Ng T.P. (2015). Objective and subjective measures of neighborhood environment (NE): Relationships with transportation physical activity among older persons. Int. J. Behav. Nutr. Phys. Act..

[31] Cerin E., Leslie E., Owen N., Bauman A. (2008). An Australian version of the Neighbourhood Environment Walkability Scale: Construct and factorial validity. Meas. Phys. Educ. Exerc. Sci..

[32] Cerin E., Sit C.H., Cheung M.C., Ho S.Y., Lee L.C., Chan W.M. (2010). Reliable and valid NEWS for Chinese seniors: Measuring perceived neighborhood attributes related to walking. Int. J. Behav. Nutr. Phys. Act..

[33] Osypuk T.L., Diez Roux A.V., Hadley C., Kandula N.R. (2009). Are immigrant enclaves healthy places to live? The Multi-ethnic Study of Atherosclerosis. Soc. Sci. Med..

[34] Li Y., Kao D., Dinh T.Q. (2015). Correlates of neighborhood environment with walking among older Asian Americans. J. Aging Health.

[35] Mackenbach J.D., Lakerveld J., van Lenthe F.J., Bardos H., Glonti K., Compernolle S., De Bourdeaudhuij I., Oppert J.M., Roda C., Rutter H. (2016). Exploring why residents of socioeconomically deprived neighbourhoods have less favourable perceptions of their neighbourhood environment than residents of wealthy neighbourhoods. Obes. Rev..

[36] Macniven R., Richards J., Gubhaju L., Joshy G., Bauman A., Banks E., Eades S. (2016). Physical activity, healthy lifestyle behaviors, neighborhood environment characteristics and social support among Australian Aboriginal and non-Aboriginal adults. Prev. Med. Rep..

[37] Lawton J., Ahmad N., Hanna L., Douglas M., Hallowell N. (2006). ‘I can’t do any serious exercise’: Barriers to physical activity amongst people of Pakistani and Indian origin with Type 2 diabetes. Health Educ. Res..

[38] Font J., Mendez M. (2013). Surveying Ethnic Minorities and Immigrant Populations—Methodological Challenges and Research Strategies.

[39] United Nations Development Programme (UNDP), HelpAge International, AARP (2017). Ageing, Older Persons and the 2030 Agenda for Sustainable Development.

[40] Simon-Davies J. Population and Migration Statistics in Australia. Research Papers Series 2018–19. https://parlinfo.aph.gov.au/parlInfo/download/library/prspub/6377182/upload_binary/6377182.pdf.

[41] Sarich P.E., Ding D., Sitas F., Weber M.F. (2015). Co-occurrence of chronic disease lifestyle risk factors in middle-aged and older immigrants: A cross-sectional analysis of 264,102 Australians. Prev. Med..

[42] Cerin E., Barnett A., Sit C.H., Cheung M.C., Lee L.C., Ho S.Y., Chan W.M. (2011). Measuring walking within and outside the neighborhood in Chinese elders: Reliability and validity. BMC Public Health.

[43] Cerin E., Zhang C.J., Barnett A., Sit C.H., Cheung M.M., Johnston J.M., Lai P.C., Lee R.S. (2016). Associations of objectively-assessed neighborhood characteristics with older adults’ total physical activity and sedentary time in an ultra-dense urban environment: Findings from the ALECS study. Health Place.

[44] Nathan A., Wood L., Giles-Corti B. (2014). Exploring socioecological correlates of active living in retirement village residents. J. Aging Phys. Act..

[45] Gilbert P.A., Khokhar S. (2008). Changing dietary habits of ethnic groups in Europe and implications for health. Nutr. Rev..

[46] Cacioppo J.T., Hughes M.E., Waite L.J., Hawkley L.C., Thisted R.A. (2006). Loneliness as a specific risk factor for depressive symptoms: Cross-sectional and longitudinal analyses. Psychol. Aging.

[47] Lai D.W., Chau S.B. (2007). Effects of service barriers on health status of older Chinese immigrants in Canada. Soc. Work.

[48] Veninga J. (2006). Social capital and health: Maximizing the benefits. Health Policy Res. Bull..

[49] Frank L.D., Sallis J.F., Saelens B.E., Leary L., Cain K., Conway T.L., Hess P.M. (2010). The development of a walkability index: Application to the Neighborhood Quality of Life Study. Br. J. Sports Med..

[50] Cerin E., Sit C.H., Barnett A., Johnston J.M., Cheung M.C., Chan W.M. (2014). Ageing in an ultra-dense metropolis: Perceived neighbourhood characteristics and utilitarian walking in Hong Kong elders. Public Health Nutr..

[51] Ma X., Barnes T.L., Freedman D.A., Bell B.A., Colabianchi N., Liese A.D. (2013). Test-retest reliability of a questionnaire measuring perceptions of neighborhood food environment. Health Place.

[52] Cochrane T., Yu Y., Davey R., Cerin E., Cain K.L., Conway T.L., Kerr J., Frank L.D., Chapman J.E., Adams M.A. (2019). Associations of built environment and proximity of food outlets with weight status: Analysis from 14 cities in 10 countries. Prev. Med..

[53] De Bourdeaudhuij I., Van Dyck D., Salvo D., Davey R., Reis R.S., Schofield G., Sarmiento O.L., Mitas J., Christiansen L.B., MacFarlane D. (2015). International study of perceived neighbourhood environmental attributes and Body Mass Index: IPEN Adult study in 12 countries. Int. J. Behav. Nutr. Phys. Act..

[54] Richard L., Gauvin L., Gosselin C., Laforest S. (2009). Staying connected: Neighbourhood correlates of social participation among older adults living in an urban environment in Montreal, Quebec. Health Promot. Int..

[55] Levasseur M., Naud D., Bruneau J.F., Genereux M. (2020). Environmental Characteristics Associated with Older Adults’ Social Participation: The Contribution of Sociodemography and Transportation in Metropolitan, Urban, and Rural Areas. Int. J. Environ. Res. Public Health.

[56] Zhang C.J.P., Barnett A., Johnston J.M., Lai P.C., Lee R.S.Y., Sit C.H.P., Cerin E. (2019). Objectively-Measured Neighbourhood Attributes as Correlates and Moderators of Quality of Life in Older Adults with Different Living Arrangements: The ALECS Cross-Sectional Study. Int. J. Environ. Res. Public Health.

[57] Boyko C.T., Cooper R. (2011). Clarifying and reconceptualising density. J. Prog. Plan..

[58] Richardson H.W., Chang-Hee C.B., Baxamusa M., Jenks M., Burgess R. (2000). Compact cities in developing countries: Assessment and implications. Compact City: Sustainable Urban Forms for Developing Countries.

[59] Needham C., Orellana L., Allender S., Sacks G., Blake M.R., Strugnell C. (2020). Food Retail Environments in Greater Melbourne 2008–2016: Longitudinal Analysis of Intra-City Variation in Density and Healthiness of Food Outlets. Int. J. Environ. Res. Public Health.

[60] Cheng Y.H., Chou K.L., MacFarlane D.J., Chi I. (2007). Patterns of physical exercise and contributing factors among Hong Kong older adults. Hong Kong Med. J..

[61] Cerin E., Sit C.H., Zhang C.J., Barnett A., Cheung M.M., Lai P.C., Johnston J.M., Lee R.S. (2016). Neighbourhood environment, physical activity, quality of life and depressive symptoms in Hong Kong older adults: A protocol for an observational study. BMJ Open.

[62] Hovell M.F., Sallis J.F., Hofstetter C.R., Spry V.M., Faucher P., Caspersen C.J. (1989). Identifying correlates of walking for exercise: An epidemiologic prerequisite for physical activity promotion. Prev. Med..

[63] Benjamins M.R., Musick M.A., Gold D.T., George L.K. (2003). Age-related declines in activity level: The relationship between chronic illness and religious activities. J. Gerontol. B Psychol. Sci. Soc. Sci..

[64] Martinez I.L., Kim K., Tanner E., Fried L.P., Seeman T. (2009). Ethnic and class variations in promoting social activities among older adults. Act. Adapt. Aging.

[65] Jansen D.A. (2005). Perceived barriers to participation in mentally restorative activities by community dwelling elders. Act. Adapt. Aging.

[66] Howat P., Iredell H., Grenade L., Nedwetzky A., Collins J. (2004). Reducing social isolation amongst older people—Implications for health professionals. Geriaction.

[67] Van Dyck D., Cerin E., Conway T.L., De Bourdeaudhuij I., Owen N., Kerr J., Cardon G., Sallis J.F. (2014). Interacting psychosocial and environmental correlates of leisure-time physical activity: A three-country study. Health Psychol..

[68] Kearney J.M., McElhone S. (1999). Perceived barriers in trying to eat healthier—Results of a pan-EU consumer attitudinal survey. Br. J. Nutr..

[69] Tourangeau R., Jabine T.B. (1984). Cognitive science and survey methods. Cognitive Aspects of Survey Methodology: Building a Bridge between Disciplines.

[70] Suen Y.N., Cerin E., Mellecker R.R. (2014). Development and reliability of a scale of physical-activity related informal social control for parents of Chinese pre-schoolers. Int. J. Behav. Nutr. Phys. Act..

[71] Landis J.R., Koch G.G. (1977). The measurement of observer agreement for categorical data. Biometrics.

[72] Cerin E., Conway T.L., Barnett A., Smith M., Veitch J., Cain K.L., Salonna F., Reis R.S., Molina-Garcia J., Hinckson E. (2019). Development and validation of the neighborhood environment walkability scale for youth across six continents. Int. J. Behav. Nutr. Phys. Act..

[73] Da W.W., Garcia A. (2015). Later life migration: Sociocultural adaptation and changes in quality of life at settlement among recent older Chinese immigrants in Canada. Act. Adapt. Aging.

[74] Ip D., Lui C.W., Chui W.H. (2007). Veiled entrapment: A study of social isolation of older Chinese migrants in Brisbane, Queensland. Ageing Soc..

[75] van der Greft S., Droogleever Fortuijn J. (2017). Multiple disadvantage of older migrants and native Dutch older adults in deprived neighbourhoods in Amsterdam, the Netherlands: A life course perspective. GeoJournal.

[76] O’Neil K., Tienda M. (2014). Age at Immigration and the Incomes of Older Immigrants, 1994–2010. J. Gerontol. Ser. B.

[77] Angel R.J., Angel J.L., Lee G.Y., Markides K.S. (1999). Age at migration and family dependency among older Mexican immigrants: Recent evidence from the Mexican American EPESE. Gerontologist.

[78] Alcorn T., Ouyang Y. (2012). China’s invisible burden of foodborne illness. Lancet.

[79] Liu X. (2014). International perspectives on food safety and regulations—A need for harmonized regulations: Perspectives in China. J. Sci. Food Agric..

[80] Satia-Abouta J., Patterson R.E., Kristal A.R., Teh C., Tu S.P. (2002). Psychosocial predictors of diet and acculturation in Chinese American and Chinese Canadian women. Ethn. Health.

[81] Bowling A. (2005). Mode of questionnaire administration can have serious effects on data quality. J. Public Health.

[82] Chu A.H., Ng S.H., Koh D., Muller-Riemenschneider F. (2015). Reliability and Validity of the Self- and Interviewer-Administered Versions of the Global Physical Activity Questionnaire (GPAQ). PLoS ONE.

[83] Chu A.H.Y., Ng S.H.X., Koh D., Muller-Riemenschneider F. (2018). Domain-Specific Adult Sedentary Behaviour Questionnaire (ASBQ) and the GPAQ Single-Item Question: A Reliability and Validity Study in an Asian Population. Int. J. Environ. Res. Public Health.

[84] Jafarpour A., Spiers H. (2017). Familiarity expands space and contracts time. Hippocampus.

[85] van de Ven N., van Rijswijk L., Roy M.M. (2011). The return trip effect: Why the return trip often seems to take less time. Psychon. Bull. Rev..

[86] Cerin E., Leslie E., Sugiyama T., Owen N. (2010). Perceived barriers to leisure-time physical activity in adults: An ecological perspective. J. Phys. Act. Health.

[87] Cerin E., Van Dyck D., Zhang C.J.P., Van Cauwenberg J., Lai P.C., Barnett A. (2020). Urban environments and objectively-assessed physical activity and sedentary time in older Belgian and Chinese community dwellers: Potential pathways of influence and the moderating role of physical function. Int. J. Behav. Nutr. Phys. Act..

[88] The Government of Hong Kong Hong Kong—The Facts. https://www.gov.hk/en/about/abouthk/facts.htm..

[89] Australian Bureau of Statistics (2018). Greater Melbourne (GCCSA) (2GMEL) Population Density at 30 June 2016.

[90] Cerin E., Barnett A., Chaix B., Nieuwenhuijsen M.J., Caeyenberghs K., Jalaludin B., Sugiyama T., Sallis J.F., Lautenschlager N.T., Ni M.Y. (2020). International Mind, Activities and Urban Places (iMAP) study: Methods of a cohort study on environmental and lifestyle influences on brain and cognitive health. BMJ Open.

[91] Cerin E., Chan K.W., Macfarlane D.J., Lee K.Y., Lai P.C. (2011). Objective assessment of walking environments in ultra-dense cities: Development and reliability of the Environment in Asia Scan Tool--Hong Kong version (EAST-HK). Health Place.

[92] Numbeo Crime Index by City 2019. https://www.numbeo.com/crime/rankings.jsp?title=2019.

[93] Cerin E., Sit C.H.P., Barnett A., Huang W.Y.J., Gao G.Y., Wong S.H.S., Sallis J.F. (2017). Reliability of self-report measures of correlates of obesity-related behaviours in Hong Kong adolescents for the iHealt(H) and IPEN adolescent studies. Arch. Public Health.

