# Supplementary material for: Development of Measures of Perceived Neighborhood Environmental Attributes Influencing, and Perceived Barriers to Engagement in, Healthy Behaviors for Older Chinese Immigrants to Australia
Source: Int J Environ Res Public Health. 2021 Apr 24;18(9):4531. doi: 10.3390/ijerph18094531 (PMC8123107; doi:10.3390/ijerph18094531)
Supplement: Supplementary file 1 [file ijerph-18-04531-s001.zip › ijerph-1179014-SI.pdf]

Table S1. Questionnaires of perceived neighbourhood attributes associated with, and perceived barriers to engagement in, physical activity, healthy eating and socialising appropriate for older Chinese immigrants to Australia: detailed description of modifications based on findings from cognitive interviews

Subscales / *items*	**Modifications based on results of cognitive interviews**
**Questionnaire: Neighbourhood Environment for Healthy Ageing – Chinese Immigrants to Australia**	
General instructions	Added to front page:<br>"*Please remember that there are no right or wrong answers*"<br>"*Please keep in mind that the questions are about Australia and the current situation in your neighbourhood*".<br>Added to each subscale:<br>"*Please put a check mark "√" next to your answer (Please only use one check mark "√" for each question).*"<br>Added to all subscales except for Residential density and Land use diversity"<br>*"Neighbourhood or 'easy to walk' means within 1km or 10-15 minute walking distance from where you live."*<br>*"The statements below are about Australia and the current situation in your neighbourhood."*<br>*"Agree - means that you agree that the statement is true."*<br>*"Disagree - means that you do not think that the statement is true."*
Residential density	Added instructions:<br>*"ATTENTION PLEASE! Neighbourhood means within 1 km or 10-15 minute walking distance from where you live."*<br>Clarified in each item that *"your neighbourhood"* means *"within 1km or 10-15 minute 1km walking distance"*.<br>Provided an example (picture) of a single-family residence.<br>Provided examples (pictures) of a multi-family house.
Land use diversity	Replaced instructions:<br>*"Please put one check mark (√) for each business or facility"* with *"Please put a check mark "√" next to your answer (Please only use one check mark "√" for each question)"*.<br>Added a new item: *"Two-dollar shop"*<br>Provided examples/explanations for five items:<br>*"Hardware store (e.g., Bunnings, Masters)"*<br>*"Chained Western or Chinese fast-food restaurants (e.g. MacDonald, KFC, Pizza Hut)"*<br>*"Western non-fast-food restaurant (sit-down restaurants that employ professional staff or chefs. They are not fast-food restaurant or coffee shops)"*<br>*"Coffee shop (it sells coffee and snacks)"*<br>*"Religious places (e.g., church, temple)"*<br>Reworded item:<br>*"Own garden / vegetable patch"* to *"Places where you can grow vegetables and fruit (e.g., own garden or public garden)"*
General access to services	Clarified in each item that the statement referred to an area *"within 1km or 10-15 minutes walking distance"* from home.<br>Replaced rating scale anchors *"somewhat agree"* and *"somewhat disagree"* with *"agree"* and *"disagree"*. Added *"unsure"* as an additional response options (between *"disagree"* and *"agree"*).
Recreational facilities	Clarified in each item that the statement referred to an area *"within 1km or 10-15 minutes walking distance"* from home.<br>Replaced rating scale anchors *"somewhat agree"* and *"somewhat disagree"* with *"agree"* and *"disagree"*. Added *"unsure"* as an additional response options (between *"disagree"* and *"agree"*).<br>Added the response option "*Not applicable*" to the following items:<br>*"The recreational facilities in my neighbourhood (within 1km or 10-15 minute walking distance) are affordable. (If you do not have any recreational facilities in your neighbourhood, please use "Not applicable")"*<br>*"Recreational centres in my neighbourhood (within 1km or 10-15 minute walking distance)*

	provide a variety of activities for the elderly. (<u>If you don't have any recreational centres in your neighbourhood, please use "Not applicable"</u>)<br>*"Recreational centres in my neighbourhood (within 1km or 10-15 minute walking distance) have Chinese-speaking instructors and volunteers. (<u>If you don't have any recreational centres in your neighbourhood, please use "Not applicable"</u>.)"*
Physical food environment	Clarified in each item that the statement referred to an area *"within 1km or 10-15 minutes walking distance"* from home.<br>Replaced rating scale anchors *"somewhat agree"* and *"somewhat disagree"* with *"agree"* and *"disagree"*. Added *"unsure"* as an additional response options (between *"disagree"* and *"agree"*).<br>Provided explanation for eight items:<br>*"There are many grocery shops (<u>including Chinese, Western or from other countries</u>) in my neighbourhood (within 1km or 10-15 minutes walking distance)"*<br>*"Healthy foods cost too much (<u>e.g., vegetables, fruit, foods that do not contain a lot of fat, salt or added sugar</u>)".*<br>*"There are some food shops in my neighbourhood (within 1km or 10-15 minutes walking distance) that have an organic food section (<u>Organic food means produce grown without using synthetic pesticides or artificial fertilisers and are not genetically modified</u>). (<u>If you do not have any food shop in your neighbourhood, please chose "Not applicable"</u>)".*<br>For five items describing the products sold in or practices of shops in the neighbourhood the following response option was given:<br>*(<u>If you do not have XX shop in your neighbourhood, please choose "Not applicable"</u>).*
Destinations for socialising	When appropriate, clarified in item that the statement referred to an area *"within 10-15 minutes or 1km walking distance"* from home.<br>Replaced rating scale anchors *"somewhat agree"* and *"somewhat disagree"* with *"agree"* and *"disagree"*. Added *"unsure"* as an additional response options (between *"disagree"* and *"agree"*).
Transportation	When appropriate, clarified in item that the statement referred to an area *"within 1km or 10-15 minutes walking distance"* from home.<br>Replaced rating scale anchors *"somewhat agree"* and *"somewhat disagree"* with *"agree"* and *"disagree"*. Added *"unsure"* as an additional response options (between *"disagree"* and *"agree"*).<br>Added the response option "*Not applicable*" to the following items:<br>*"Parking is too expensive"*; *"Public transport services are too expensive"*<br>Expanded the item *"It is easy to walk to a transit stop (bus, tram) from my home"* into three sub-items for specific modes of transport:<br>*"It is easy to walk to the following transit stop from home (within 1km or 10-15 minutes walking distance).*<br>*<u>Bus stop</u>*<br>*<u>Tram stop</u>*<br>*<u>Train stop</u>"*<br>Provided further explanation, added the response option "*Not applicable*" and expanded the item *"Public transport is easy to use"* into three sub-items for specific modes of transport (bus, tram and train):<br>*"Public transport in my neighbourhood (within 1km or 10-15 minutes walking distance) is easy to use (<u>e.g., easy to swipe Myki, not crowded or easy to get on and off</u>).*<br>*<u>Bus/bus stop</u>*<br>*<u>Tram/tram stop</u>*<br>*<u>Train/train stop</u>"*<br>Provided further explanation, added the response option "*Not applicable*" and expanded the item *"Buses, trains and trams provide clear information on the next stop (stop name or number) so that I know when I need to get off"* into three sub-items for specific modes of transport (bus, tram and train):<br>*"Public transport in my neighbourhood (within 1km or 10-15 minutes walking distance) provides clear information on the next stop (stop name or number) so that I know when to get off (<u>if you do not have a specific type of public transport in your neighbourhood, please use "Not applicable"</u>).*

	Bus<br>*Tram*<br>*Train”*<br>Provided further explanation, added the response option “*Not applicable*”, changed and expanded the item *“Public transport in my area is infrequent”* into three sub-items for specific modes of transport (bus, tram and train):<br>*“Public transport in my neighbourhood (within 1km or 10-15 minutes walking distance) is frequent (if you do not have a specific type of public transport in your neighbourhood, please use “Not applicable”).*<br>*Bus*<br>*Tram*<br>*Train”*<br>Changed and expanded the item *“Transit stop and public transport signage is poor (e.g., cannot read transit stop number or bus number)”* into three sub-items for specific modes of transport (bus, tram and train):<br>*“Transit stop and public transport signage is clear (e.g., transit stop number or bus number is easy to read).*<br>*Bus*<br>*Tram*<br>*Train”*
Physical barriers to walking	Clarified in each item that the statement referred to an area *“within 1km or 10-15 minutes walking distance”* from home.<br>Replaced rating scale anchors *“somewhat agree”* and *“somewhat disagree”* with *“agree”* and *“disagree”*. Added *“unsure”* as an additional response options (between *“disagree”* and *“agree”*).
Street connectivity	Clarified in each item that the statement referred to an area *“within 1km or 10-15 minutes walking distance”* from home.<br>Replaced rating scale anchors *“somewhat agree”* and *“somewhat disagree”* with *“agree”* and *“disagree”*. Added *“unsure”* as an additional response options (between *“disagree”* and *“agree”*).
Infrastructure for pedestrians	When appropriate, clarified in item that the statement referred to an area *“within 1km or 10-15 minutes walking distance”* from home.<br>Replaced rating scale anchors *“somewhat agree”* and *“somewhat disagree”* with *“agree”* and *“disagree”*. Added *“unsure”* as an additional response options (between *“disagree”* and *“agree”*).
Indoor places for walking	Clarified in each item that the statement referred to an area *“within 1km or 10-15 minutes walking distance”* from home.<br>Replaced rating scale anchors *“somewhat agree”* and *“somewhat disagree”* with *“agree”* and *“disagree”*. Added *“unsure”* as an additional response options (between *“disagree”* and *“agree”*).
Aesthetics	Clarified in each item that the statement referred to an area *“within 1km or 10-15 minutes walking distance”* from home.<br>Replaced rating scale anchors *“somewhat agree”* and *“somewhat disagree”* with *“agree”* and *“disagree”*. Added *“unsure”* as an additional response options (between *“disagree”* and *“agree”*).
Traffic and road hazards	Clarified in each item that the statement referred to an area *“within 1km or 10-15 minutes walking distance”* from home.<br>Replaced rating scale anchors *“somewhat agree”* and *“somewhat disagree”* with *“agree”* and *“disagree”*. Added *“unsure”* as an additional response options (between *“disagree”* and *“agree”*).<br>Provided further explanation for the item *“The streets and sidewalks in my neighbourhood are often slippery”*:<br>*“The streets and sidewalks in my neighbourhood (within 1km or 10-15 minutes walking distance) are often slippery (e.g., when it rains, the floor becomes slippery, or people often throw water on the street)”*.
Traffic speed	Clarified in each item that the statement referred to an area *“within 1km or 10-15 minutes walking distance”* from home.

	Replaced rating scale anchors *"somewhat agree"* and *"somewhat disagree"* with *"agree"* and *"disagree"*. Added *"unsure"* as an additional response options (between *"disagree"* and *"agree"*).
Social disorder / littering	Clarified in each item that the statement referred to an area *"within 1km or 10-15 minutes walking distance"* from home.<br>Replaced rating scale anchors *"somewhat agree"* and *"somewhat disagree"* with *"agree"* and *"disagree"*. Added *"unsure"* as an additional response options (between *"disagree"* and *"agree"*).
Crime	Clarified in each item that the statement referred to an area *"within 1km or 10-15 minutes walking distance"* from home.<br>Replaced rating scale anchors *"somewhat agree"* and *"somewhat disagree"* with *"agree"* and *"disagree"*. Added *"unsure"* as an additional response options (between *"disagree"* and *"agree"*).
Safety – presence of people	Clarified in each item that the statement referred to an area *"within 1km or 10-15 minutes walking distance"* from home.<br>Replaced rating scale anchors *"somewhat agree"* and *"somewhat disagree"* with *"agree"* and *"disagree"*. Added *"unsure"* as an additional response options (between *"disagree"* and *"agree"*).
Social environment and communication – physical activity	Clarified in each item that the statement referred to an area *"within 1km or 10-15 minutes walking distance"* from home.<br>Replaced rating scale anchors *"somewhat agree"* and *"somewhat disagree"* with *"agree"* and *"disagree"*. Added *"unsure"* as an additional response options (between *"disagree"* and *"agree"*).<br>Provided further explanation, changed and added the response option "*Not applicable*" to the item "*Our local volunteer organisations organise outdoor physical activity sessions":*<br>*"Volunteer organisations in my neighbourhood (within 1km or 10-15 minutes walking distance) organise physical activity sessions (e.g., walking activities) (if you do not have any volunteer organisations in your neighbourhood, please use "Not applicable")."*
Social environment and communication – healthy eating<br>-	When appropriate, clarified in item that the statement referred to an area *"within 1km or 10-15 minutes walking distance"* from home.<br>Replaced rating scale anchors *"somewhat agree"* and *"somewhat disagree"* with *"agree"* and *"disagree"*. Added *"unsure"* as an additional response options (between *"disagree"* and *"agree"*).<br>Provided further explanation for the item "*There are people in my neighbourhood who I can talk to about healthy eating":*<br>*"There are people (anyone, including family, friends, neighbours, professionals, etc.) in my neighbourhood (within 1km or 10-15 minutes walking distance) who I can talk to about healthy eating"*.<br>Changed the item "*Our local Chinese newspaper / magazine publishes information on healthy eating"* to:<br>"*Our Australian local Chinese newspaper / magazine publishes information on healthy eating."*<br>Changed the item "*Our local community organises talks on healthy eating in Chinese"* to:<br>"*Our neighbourhood (within 1km or 10-15 minutes walking distance) organises talks on healthy eating in Chinese".*
Social environment and communication – socialising	Clarified in each item that the statement referred to an area *"within 1km or 10-15 minutes walking distance"* from home.<br>Replaced rating scale anchors *"somewhat agree"* and *"somewhat disagree"* with *"agree"* and *"disagree"*. Added *"unsure"* as an additional response options (between *"disagree"* and *"agree"*).<br>Added the response option "*Not applicable*" to the item "*Clinics, shops and other services in my neighbourhood (within 1km or 10-15 minutes walking distance) have staff who can speak English and Chinese (if you do not have clinics, shops or other services in your neighbourhood please use "Not applicable")."*

Questionnaire: Perceived barriers to engaging in health-enhancing behaviours

Perceived barriers to leisure-time physical activity

Added to instructions: *"This section is about regular exercise, including walking for recreational purposes (e.g., walking in leisure time, Tai-Chi, etc.)."*

Changed instructions and item structure from *"How often do the following prevent you from getting regular exercise including walking in your leisure time (do not include walking to go to/from places)? (Please circle the most appropriate answer – one response per question)*

Fear or injury	*Never*	*Rarely*	*Sometimes*	*Often*	*Very often*
	1	2	3	4	5 "

to:

"Do the following reasons prevent you from getting regular exercise including walking for recreational purposes (e.g., walking in leisure time, Tai-Chi, etc.)?
Please circle the most appropriate answer – one response per question.
Example: "Fear of injury [often] prevents me from getting regular exercise including walking for recreational purposes."

Never	*Rarely*	*Sometimes*	*Often*	*Very often*
1	2	3	4	5"

Provided further explanation for three items:
"Self-conscious about my looks" (e.g., dislike being sweaty or worry about my own weight) …
"Lack of self-discipline" (e.g., feeling lazy and do not want to do the pre-planned physical activity) …
"Air pollution in Australia" …

Perceived barriers to walking for transport

Added to instructions: *"This section is about regular walking for transport to go to/from places (e.g., walking to go to work or to get groceries)."*

Changed instructions and item structure from *"How often do the following prevent you from walking for transport (walking to go to/from places)? (Please circle the most appropriate answer – one response per question)*

Lack of energy	*Never*	*Rarely*	*Sometimes*	*Often*	*Very often*
	1	2	3	4	5 "

to:

"Do the following reasons prevent you from walking for transport to go to/from places (e.g., walking to go to work or to get groceries)?
Please circle the most appropriate answer – one response per question.
Example: "Lack of energy" [sometimes] prevents me from walking for transport to get to/from places."

Never	*Rarely*	*Sometimes*	*Often*	*Very often*
1	2	3	4	5"

Provided further explanation for two items:
"Traffic hazards" (e.g., nearby streets have lots of traffic or lack pedestrian crossings) …
"Air pollution in Australia" …

Perceived barries to healthy eating

Changed instructions and item structure from *"How often the following are barriers to you eating a healthy diet? (Please circle the most appropriate answer – one response per question)*

Grocery stores are too far away from home	*Never*	*Rarely*	*Sometimes*	*Often*	*Very often*
	1	2	3	4	5 "

to:

"Do the following reasons prevent you from eating a healthy diet (e.g., vegetables, fruits, foods that do not contain a lot of fat, salt and added sugar)?
Please circle the most appropriate answer – one response per question.
Example: "Grocery stores being too far away from home" [very often] prevents me from eating a healthy diet."

Never	*Rarely*	*Sometimes*	*Often*	*Very often*

	1 2 3 4 5”<br><br>Provided further explanation for eight items:<br>*“Lack of information about a healthy diet in Chinese” (e.g., lack of Chinese newspaper, website, etc.) …*<br>*All items mentioning healthy foods had the following added “(e.g., vegetables, fruits, foods that do not contain a lot of fat, salt and added sugar)” …*<br>*“Eating out” ______ prevents me from eating a healthy diet (e.g., If when I eat out, I always choose healthy food options, then “eating out” never prevents me from eating a healthy diet).*<br>Added the response option “*Not applicable*” to the items:<br>*“Lack of time to prepare healthy foods” … (if you do not cook, please choose “Not applicable”)*<br>*“Lack of energy/motivation to prepare healthy foods” … (if you do not cook, please choose “Not applicable”)*<br>*“Lack of skills to plan, shop for or cook healthy foods” … (if you do not cook, please choose “Not applicable”)*<br>*“Lack of friends’ support for eating a healthy diet” … (if you do not have friends, please choose “Not applicable”)*<br>*“Lack of partner’s support for eating a healthy diet” … (if you do not have a partner, please choose “Not applicable”)*<br>*“Lack of children’s support for eating a healthy diet” … (if you do not have children, please choose “Not applicable”)*
Perceived barriers to socialising	Changed instructions and item structure from *“How often the following are barriers to you socialising? (Please circle the most appropriate answer – one response per question)*<br>*Lack of Chinese-speaking people Never Rarely Sometimes Often Very often*<br>1 2 3 4 5 ”<br>to:<br>*“Do the following reasons prevent you from socialising?*<br>*Please circle the most appropriate answer – one response per question.*<br>*Example: “Lack of Chinese-speaking people” [very often] prevents me from socialising.”*<br><br>*Never Rarely Sometimes Often Very often*<br>1 2 3 4 5”<br>Added the response option “*Not applicable*” to the items:<br>*“Lack of partner’s support” … (if you do not have a partner, please choose “Not applicable”)*<br>*“Lack of children’s support” … (if you do not have children, please choose “Not applicable”)*

Table S2. Descriptive statistics, test-retest reliability and internal consistency of measures of perceived neighbourhood environmental attributes influencing, and perceived barriers to engagement in, healthy behaviours for older Chinese immigrants to Australia

**Measure [theoretical range]**	M (SD) [assessment 1]	M (SD) [assessment 2]	ICC (95% CI)
Questionnaire: Neighbourhood Environment for Healthy Ageing – Chinese Immigrants to Australia			
Residential density [6-789]			
1) Detached single-family houses	3.27 (0.97)	3.23 (1.00)	0.94 (0.90, 0.97)
2) Multi-family houses	2.46 (0.80)	2.38 (0.80)	0.88 (0.80, 0.93)
3) Apartments (4-6 stories)	1.48 (0.70)	1.44 (0.70)	0.96 (0.93, 0.98)
4) Apartments (7-12 stories)	1.25 (0.44)	1.27 (0.45)	0.95 (0.92, 0.97)
5) Apartments (13-20 stories)	1.04 (0.19)	1.08 (0.27)	0.65 (0.45, 0.78)
6) Apartments (>20 stories)	1.04 (0.19)	1.04 (0.19)	1.00 (N/A)
Land use diversity (distance to destinations) [1-5]			
1) Convenience / small grocery shop	2.67 (1.12)	2.67 (1.08)	0.97 (0.95, 0.98)
2) Supermarket	3.25 (1.08)	3.25 (1.01)	0.89 (0.82, 0.94)
3) Two-dollar shop	3.77 (1.21)	3.81 (1.14)	0.56 (0.34, 0.72)
4) Fresh food market	3.31 (1.16)	3.40 (1.12) *	0.52 (0.26, 0.70)
5) Hardware store	4.77 (0.58)	4.69 (0.61)	0.79 (0.65, 0.87)
6) Clothing & show shop	3.77 (1.00)	3.77 (1.00)	0.92 (0.87, 0.96)
7) Pharmacy / drugstore	3.38 (0.97)	3.30 (0.96)	0.92 (0.86, 0.95)
8) Book / stationary shop	3.60 (1.09)	3.52 (1.09) *	0.44 (0.20, 0.63)
9) Video / audio shop	3.96 (1.07)	3.85 (1.04) *	0.53 (0.28, 0.71)
10) Library	3.79 (1.11)	3.73 (1.25)	0.92 (0.87, 0.96)
11) Laundry / dry cleaner	3.48 (1.08)	3.35 (1.23) *	0.48 (0.24, 0.66)
12) Salon / barber shop	3.29 (1.02)	3.17 (0.94)	0.90 (0.83, 0.94)
13) Bank / credit union	3.60 (1.05)	3.50 (1.16) *	0.68 (0.40, 0.83)
14) Post office	3.33 (0.98)	3.31 (1.06)	0.92 (0.86, 0.95)
15) Doctor / clinical service	3.54 (1.21)	3.54 (1.14)	0.95 (0.91, 0.97)
16) Primary school	2.83 (1.25)	2.79 (1.22)	0.64 (0.44, 0.78)
17) Nursery school	2.83 (1.40)	2.77 (1.42)	0.72 (0.56, 0.83)
18) Chained Western or Chinese fast-food restaurant	3.25 (1.08)	3.17 (1.02)	0.53 (0.29, 0.70)
19) Chinese coffee shop or noodle shop	3.60 (0.91)	3.60 (0.87)	0.90 (0.84, 0.94)
20) Chinese non-fast food restaurant	3.73 (1.22)	3.77 (1.15)	0.72 (0.56, 0.83)
21) Western non-fast food restaurant	3.83 (1.15)	3.79 (1.05)	0.55 (0.33, 0.72)
22) Coffee shop	3.29 (1.26)	3.27 (1.32)	0.44 (0.19, 0.63)
23) Community centre	3.50 (1.11)	3.42 (1.21)	0.94 (0.90, 0.97)
24) Elderly centre	4.04 (0.99)	3.92 (1.08)	0.95 (0.90, 0.97)
25) Park	2.80 (1.38)	2.84 (1.34)	0.64 (0.44, 0.78)
26) Gym or fitness facility	3.40 (1.14)	3.33 (1.31)	0.89 (0.81, 0.93)
27) Swimming pool	4.50 (0.85)	4.40 (0.93) *	0.36 (0.02, 0.61)
28) Religious places	3.50 (1.13)	3.46 (1.20)	0.93 (0.88, 0.96)
29) Public toilet	3.96 (1.19)	3.88 (1.31) *	0.37 (0.12, 0.58)
30) Bakery / cake shop	3.42 (1.19)	3.38 (1.09)	0.63 (0.43, 0.77)
31) Bus stop	1.94 (0.89)	1.77 (0.92) *	0.85 (0.74, 0.92)
32) Tram stop	4.46 (0.96)	4.46 (1.00)	0.96 (0.93, 0.98)
33) Train stop	3.92 (1.01)	3.88 (1.10)	0.98 (0.97, 0.99)
34) Lottery / betting outlet	4.12 (1.04)	4.00 (1.07) *	0.44 (0.05, 0.67)
35) Place for growing vegetables and fruit	2.58 (1.88)	2.58 (1.88)	1.00 (N/A)
General access to services# [1-4]			
1) Shops within walking distance	2.81 (0.66)	2.83 (0.55)	0.71 (0.55, 0.82)
2) Shopping accessible via public transport	3.04 (0.56)	2.98 (0.50)	0.71 (0.55, 0.82)
3) Many places within walking distance	2.88 (0.55)	2.87 (0.53)	0.63 (0.44, 0.77)

Recreational facilities# [1-4]			
1) Many recreational facilities	2.56 (0.83)	2.52 (0.78)	0.97 (0.95, 0.98)
2) Affordable recreational facilities	2.46 (0.70)	2.42 (0.63)	0.49 (0.25, 0.67)
3) Activities for elderly at recreational facilities	2.69 (1.48)	2.69 (1.42)	0.81 (0.69, 0.89)
4) Recreational facilities have Chinese-speaking staff	2.58 (1.51)	2.56 (1.50)	0.79 (0.66, 0.87)
Physical food environment# [1-4]			
1) Many grocery shops	2.85 (0.75)	2.90 (0.69)	0.83 (0.73, 0.90)
2) Food outlets with strict food and hygiene policies	2.54 (0.70)	2.71 (0.78)**	0.85 (0.71, 0.92)
3) Health foods expensive	2.58 (0.64)	2.69 (0.61)	0.76 (0.61, 0.85)
4) Organic food section in food shops	2.56 (0.70)	2.52 (0.75)	0.74 (0.59, 0.84)
5) Large selection of low-fat products in shops	2.67 (0.68)	2.63 (0.63)	0.74 (0.59, 0.84)
6) High quality fresh fruit and vegetables in shops	2.73 (0.66)	2.73 (0.72)	0.89 (0.80, 0.93)
7) Chinese food labelling in shops	2.15 (0.72)	2.17 (0.71)	0.91 (0.84, 0.95)
8) Asian grocery shop in neighbourhood	2.50 (0.73)	2.44 (0.57)	0.89 (0.81, 0.93)
9) Residents can grow fruit and vegetables	2.65 (0.62)	2.77 (0.55)	0.72 (0.56, 0.83)
10) Shops selling fresh fruit and vegetable juice	2.60 (0.69)	2.65 (0.76)	0.35 (0.09, 0.57)
Destinations for socialising# [1-4]			
1) Chinese senior club in neighbourhood	2.44 (0.83)	2.40 (0.87)	0.95 (0.90, 0.97)
2) Public housing for the elderly	2.27 (0.81)	2.21 (0.72)	0.82 (0.71, 0.89)
3) Many destinations for meeting people	2.44 (0.61)	2.46 (0.67)	0.93 (0.88, 0.96)
4) Library with Chinese books/magazines	2.50 (0.78)	2.46 (0.67)	0.78 (0.65, 0.87)
5) Free Wi-Fi making it possible to keep in touch with family/friends	2.29 (0.72)	2.37 (0.77)	0.90 (0.83, 0.94)
Transportation# [1-4]			
1) Easy to walk to bus stop	3.08 (0.48)	3.12 (0.51)	0.61 (0.40, 0.75)
2) Easy to walk to tram stop	2.23 (0.83)	2.19 (0.77)	0.79 (0.66, 0.87)
3) Easy to walk to train stop	2.56 (0.80)	2.54 (0.73)	0.77 (0.65, 0.87)
4) Parking in neighbourhood is expensive	2.35 (0.62)	2.38 (0.63)	0.72 (0.56, 0.83)
5) Bus easy to use	3.12 (0.43)	3.19 (0.49) *	0.82 (0.70, 0.89)
6) Tram easy to use	2.27 (0.63)	2.33 (0.55)	0.92 (0.86, 0.95)
7) Train easy to use	2.65 (0.71)	2.67 (0.73)	0.43 (0.03, 0.69)
8) Clear information on next bus stop	2.81 (0.40)	2.85 (0.72)	0.72 (0.56, 0.83)
9) Clear information on next tram stop	2.42 (0.57)	2.38 (0.63)	0.79 (0.65, 0.87)
10) Clear information on next train stop	2.58 (0.57)	2.73 (0.77) **	0.84 (0.70, 0.91)
11) Buses are frequent	2.90 (0.69)	2.90 (0.63)	0.74 (0.58, 0.84)
12) Trams are frequent	2.25 (0.79)	2.35 (0.93)	0.88 (0.81, 0.93)
13) Trains are frequent	2.40 (0.85)	2.31 (1.02)	0.79 (0.67, 0.88)
14) Public transport is too expensive	2.58 (0.61)	2.56 (0.57)	0.68 (0.40, 0.83)
15) Bus / bus stop has clear signage	2.81 (0.56)	2.81 (0.40)	0.84 (0.73, 0.90)
16) Tram / tram stop has clear signage	2.58 (0.72)	2.54 (0.73)	0.51 (0.29, 0.69)
17) Train / train stop has clear signage	2.90 (0.72)	2.71 (0.83)	0.56 (0.35, 0.72)
18) Public transport to get to/from grocery shops	2.71 (0.61)	2.73 (0.56)	0.69 (0.51, 0.81)
Physical barriers to walking# [1-4]			
1) Hilly streets	2.65 (0.88)	2.54 (0.70)	0.79 (0.66, 0.87)
2) Major barriers to walking	2.12 (0.51)	2.08 (0.55)	0.80 (0.67, 0.88)
3) Many cul-de-sacs	2.27 (0.45)	2.27 (0.45)	1.00 (N/A)
Street connectivity# [1-4]			
1) Short distance between intersections	2.77 (0.51)	2.73 (0.45)	0.48 (0.25, 0.66)
2) Many alternative routes for getting from place to place	2.85 (0.46)	2.90 (0.53)	0.88 (0.81, 0.93)
Infrastructure for pedestrians# [1-4]			
1) Need to walk over a bridge or through	1.92 (0.55)	1.96 (0.59)	0.71 (0.54, 0.82)

tunnel			
2) Easy access to home entrance/exit	3.00 (0.63)	2.92 (0.68)	0.82 (0.71, 0.89)
3) Sidewalks on most streets	3.19 (0.49)	3.12 (0.43)	0.64 (0.44, 0.77)
4) Fence or grass/dirt strip separating traffic from sidewalks	2.75 (0.65)	2.87 (0.60) *	0.85 (0.75, 0.91)
5) Streets well-lit at night	2.52 (0.70)	2.48 (0.50)	0.85 (0.75, 0.91)
6) Crosswalks and pedestrian signals to help pedestrians cross busy roads	2.73 (0.60)	2.69 (0.78)	0.44 (0.05, 0.67)
7) Sitting facilities	2.52 (0.70)	2.40 (0.75)	0.82 (0.70, 0.89)
Indoor places for walking# [1-4]			
1) Covered sidewalks	2.21 (0.57)	2.17 (0.73)	0.87 (0.78, 0.92)
2) Indoor, air-conditioned places for walking	2.48 (0.73)	2.44 (0.78)	0.60 (0.34, 0.76)
Aesthetics# [1-4]			
1) Trees along the streets	3.07 (0.27)	3.12 (0.32)	0.78 (0.65, 0.87)
2) Many interesting things to look at	2.48 (0.64)	2.60 (0.69)	0.77 (0.65, 0.87)
3) Many attractive natural sights	2.63 (0.56)	2.58 (0.72)	0.76 (0.61, 0.85)
4) Many attractive buildings/homes	2.44 (0.63)	2.42 (0.65)	0.91 (0.84, 0.95)
Traffic and road hazards# [1-4]			
1) Motor vehicle parked on sidewalks making it difficult to walk	2.04 (0.59)	2.10 (0.53)	0.91 (0.85, 0.95)
2) Streets and sidewalks often slippery	1.92 (0.27)	1.98 (0.37)	0.73 (0.57, 0.83)
3) Level of air pollution high	1.77 (0.61)	1.88 (0.51)	0.52 (0.30, 0.69)
4) So much traffic that it is unpleasant to walk	1.90 (0.51)	1.98 (0.47)	0.56 (0.34, 0.72)
5) Parked cars that block vision and make it difficult to cross the road	2.13 (0.56)	2.17 (0.38)	0.32 (0.05, 0.54)
6) Fear of crossing the road due to many passing cars	2.08 (0.49)	2.16 (0.47)	0.69 (0.51, 0.81)
Traffic speed# [1-4]			
1) Speed of traffic usually slow	2.50 (0.58)	2.54 (0.64)	0.75 (0.35, 0.89)
2) Most drivers exceed the posted speed limits	1.98 (0.58)	2.04 (0.52)	0.78 (0.64, 0.87)
Social disorder / littering# [1-4]			
1) Animal droppings	2.08 (0.48)	2.06 (0.37)	0.73 (0.58, 0.83)
2) Many homeless people, drug addicts, prostitutes	1.67 (0.47)	1.75 (0.44)	0.63 (0.44, 0.77)
Crime# [1-4]			
1) High crime rate	1.96 (0.56)	2.06 (0.50)	0.76 (0.62, 0.86)
2) Unsafe to walk during the day	1.90 (0.50)	1.92 (0.48)	0.63 (0.44, 0.77)
3) Unsafe to walk at night	2.08 (0.71)	2.13 (0.53)	0.73 (0.57, 0.83)
Safety – Presence of people# [1-4]			
1) Walkers can be seen	2.81 (0.56)	2.75 (0.48)	0.90 (0.82, 0.94)
2) Difficult to ask for help because not many people around	2.31 (0.61)	2.37 (0.66)	0.79 (0.66, 0.87)
Social environment and communication – Physical activity# [1-4]			
1) Volunteer organisations organise outdoor physical activity sessions	2.23 (0.65)	2.21 (0.64)	0.56 (0.35, 0.72)
2) Easy to find information on health, policies and services promoting physical activity	2.06 (0.70)	2.12 (0.73)	0.76 (0.61, 0.85)
Social environment and communication – Healthy diet# [1-4]			
1) People to talk to about healthy eating	2.50 (0.75)	2.62 (0.57) *	0.77 (0.67, 0.83)
2) Local Chinese press publishes information on healthy eating	2.98 (0.50)	2.94 (0.46)	0.54 (0.32, 0.73)
3) Community organising talks on healthy eating in Chinese	2.04 (0.82)	2.08 (0.68)	0.52 (0.22, 0.75)
Social environment and communication –			

Socialising# [1-4]			
1) Free-of-charge group activities for Chinese elders	2.40 (0.77)	2.33 (0.86)	0.80 (0.67, 0.88)
2) Services have staff who can speak English and Chinese	2.62 (0.80)	2.54 (0.80)	0.48 (0.24, 0.66)
3) Formal and informal opportunities for Chinese- and English-speaking older residents	2.33 (0.81)	2.37 (0.66)	0.64 (0.44, 0.78)
4) Many Chinese-speaking residents	2.62 (0.75)	2.56 (0.61)	0.61 (0.38, 0.77)
5) Chinese-speaking policemen	1.85 (0.61)	1.88 (0.62)	0.49 (0.21, 0.69)
Questionnaire: Perceived barriers to engaging in health-enhancing behaviours			
Perceived barriers to leisure-time physical activity [1-5]			
1) Self-conscious about look	1.63 (0.77)	1.58 (0.78)	0.76 (0.61, 0.85)
2) Lack of interest in physical activity	1.96 (0.95)	1.94 (0.85)	0.85 (0.75, 0.91)
3) Lack of self-discipline	2.25 (0.90)	2.13 (0.82)	0.77 (0.63, 0.86)
4) Lack of time	2.23 (0.81)	2.20 (0.78)	0.46 (0.22, 0.65)
5) Lack of energy	2.29 (0.98)	2.35 (0.90)	0.46 (0.21, 0.65)
6) Lack of company	2.04 (0.99)	1.98 (0.92)	0.80 (0.68, 0.88)
7) Lack of enjoyment in physical activity	1.92 (0.99)	2.02 (0.92)	0.56 (0.27, 0.76)
8) Discouragement by others	1.55 (0.78)	1.54 (0.73)	0.85 (0.75, 0.91)
9) Lack of equipment	2.40 (1.18)	2. 54 (1.09)	0.61 (0.34, 0.78)
10) Lack of good weather	2.33 (0.81)	2.35 (0.86)	0.65 (0.46, 0.79)
11) Lack of skills / knowledge	2.06 (0.98)	2.10 (0.87)	0.73 (0.57, 0.84)
12) Lack of facilities, community activities or places to walk	2.46 (1.09)	2.58 (1.00)	0.84 (0.74, 0.91)
13) Lack of good health	2.65 (0.76)	2.69 (0.70)	0.68 (0.50, 0.80)
14) Fear of injury	2.38 (0.80)	2.25 (0.65)	0.59 (0.38, 0.74)
15) Air pollution	1.34 (0.52)	1.31 (0.47)	0.57 (0.35, 0.73)
16) Lack of money	1.75 (0.93)	1.73 (0.91)	0.83 (0.72, 0.90)
17) Inability to communicate with others in English	1.96 (0.88)	2.06 (1.00)	0.80 (0.67, 0.88)
18) Lack of information on community activities	2.19 (0.95)	2.15 (0.89)	0.52 (0.30, 0.70)
19) Difficulties in getting to/from recreational destinations	2.62 (1.09)	2.38 (0.91) **	0.47 (0.25, 0.66)
20) Being dependent on other family members	1.98 (0.90)	1.92 (0.84)	0.91 (0.85, 0.95)
Barriers to walking for transport			
1) Traffic hazards	1.92 (0.88)	1.79 (0.75)	0.73 (0.57, 0.84)
2) Lack of enjoyment in walking	1.79 (0.91)	1.63 (0.91)	0.82 (0.70, 0.89)
3) Physical barriers to destinations	2.12 (0.98)	2.10 (0.98)	0.50 (0.24, 0.71)
4) Lack of time	1.96 (0.82)	1.92 (0.76)	0.72 (0.56, 0.83)
5) Lack of energy	2.10 (0.93)	2.25 (0.79) *	0.56 (0.33, 0.74)
6) Lack of company	1.81 (0.81)	1.79 (0.87)	0.80 (0.67, 0.88)
7) Air pollution	1.38 (0.53)	1.35 (0.49)	0.52 (0.28, 0.69)
8) Discouragement by others	1.44 (0.57)	1.52 (0.58)	0.54 (0.31, 0.70)
9) Lack of good weather	2.17 (0.92)	2.19 (0.79)	0.83 (0.72, 0.90)
10) Lack of places to walk to	2.33 (0.88)	2.25 (1.01)	0.83 (0.72, 0.90)
11) Lack of good health	2.35 (0.86)	2.44 (0.83)	0.70 (0.57, 0.78)
12) Crime rate	2.06 (0.78)	2.02 (0.80)	0.72 (0.56, 0.83)
Perceived barriers to healthy eating [1-5]			
1) Lack of information in Chinese	2.65 (1.31)	2.62 (1.24)	0.46 (0.15, 0.69)
2) Lack of enjoyment	2.65 (1.22)	2.50 (1.22) *	0.62 (0.34, 0.78)
3) Healthy foods too expensive	2.77 (1.21)	2.85 (1.26)	0.50 (0.24, 0.71)
4) Lack of time	2.12 (1.02)	2.25 (1.06)	0.46 (0.17, 0.69)
5) Lack of energy / motivation	2.27 (1.16)	2.25 (1.14)	0.65 (0.47, 0.79)

6) Lack of skills	2.42 (1.04)	2.38 (1.05)	0.85 (0.77, 0.92)
7) Grocery shops too far away	2.38 (1.09)	2.42 (1.04)	0.60 (0.39, 0.75)
8) Eating out	2.52 (1.15)	2.42 (1.05)	0.67 (0.43, 0.81)
9) Lack of healthy options in shops	2.33 (1.00)	2.33 (1.04)	0.85 (0.76, 0.91)
10) Lack of friends' support	1.92 (0.93)	1.98 (0.92)	0.80 (0.69, 0.89)
11) Lack of partner's support	1.75 (1.05)	1.75 (1.01)	0.66 (0.42, 0.81)
12) Lack of children's support	1.75 (1.08)	1.75 (1.06)	0.87 (0.78, 0.92)
Perceived barriers to socialising [1-5]			
1) Inability to speak English	3.10 (1.39)	3.13 (1.43)	0.47 (0.22, 0.74)
2) Lack of activity centres and facilities for Chinese elderly	3.06 (1.30)	3.13 (1.27)	0.87 (0.79, 0.92)
3) Lack of good health	2.46 (0.75)	2.44 (0.77)	0.69 (0.52, 0.81)
4) Poor public transport	2.38 (0.89)	2.38 (0.99)	0.76 (0.62, 0.86)
5) Shyness	1.79 (0.72)	1.65 (0.71)	0.61 (0.41, 0.76)
6) Lack of Chinese-speaking people	2.94 (1.09)	3.00 (1.17)	0.86 (0.76, 0.92)
7) Lack of time	2.25 (0.79)	2.15 (0.80)	0.77 (0.64, 0.86)
8) Lack of motivation	2.36 (0.93)	2.46 (0.83)	0.59 (0.38, 0.74)
9) Lack of information on events and meeting groups for Chinese elderly	2.83 (1.06)	2.84 (1.07)	0.87 (0.79, 0.93)
10) Lack of partner's support	1.98 (1.08)	1.96 (0.99)	0.59 (0.28, 0.76)
11) Lack of children's support	1.87 (1.05)	1.87 (1.01)	0.45 (0.20, 0.64)

Notes. # rescaled from a 5-point to a 4-point scale to allow comparison with previous studies. * $p<.05$; ** $p<.01$

Final versions of questionnaires

Neighbourhood Environment for Healthy Ageing – Chinese Immigrants to Australia

Important instructions – please read:
In this survey, we would like to find out about the way that you perceive or think about your neighbourhood. Please remember that there are no right or wrong answers. Please keep in mind that the questions are about Australia and the current situation in your neighbourhood.

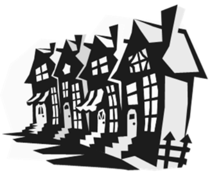

A. Types of residences in your neighbourhood

Please choose the answer that best applies to you and your neighbourhood.

Please put a check mark "√" next to your answer (Please only use <u>one</u> check mark "√" for each question).
ATTENTION PLEASE! Neighbourhood means within 1km or <u>10-15 minutes</u> walking distance from where you live.

1. How common are <u>detached single-family residences</u> in your neighbourhood (within 1km or 10-15 minutes walking distance)?

1	2	3	4	5
None	A few	Some	Most	All

For example: There is only one family living in a house on one piece of land.

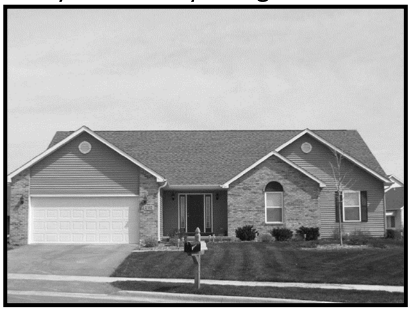

2. How common are <u>multi-family houses or apartments or condos of 1-3 stories</u> (includes apartment, townhouse, unit) in your neighbourhood (within 1km or 10-15 minutes walking distance)?

1	2	3	4	5
None	A few	Some	Most	All

For example: Multi-family sharing one piece of land.

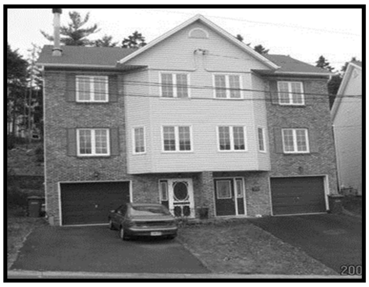 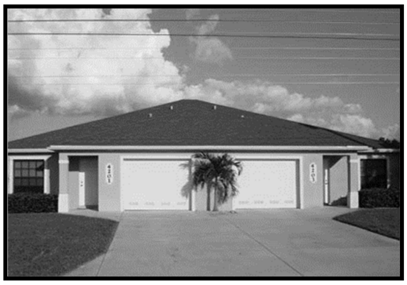

3. How common are apartments or condos of 4-6 stories in your neighbourhood (within 1km or 10-15 minutes walking distance)?

1	2	3	4	5
None	A few	Some	Most	All

4. How common are apartments or condos of 7-12 stories in your neighbourhood (within 1km or 10-15 minutes walking distance)?

1	2	3	4	5
None	A few	Some	Most	All

5. How common are apartments or condos of 13-20 stories in your neighbourhood (within 1km or 10-15 minutes walking distance)?

1	2	3	4	5
None	A few	Some	Most	All

6. How common are apartments or condos of more than 20 stories in your neighbourhood (within 1km or 10-15 minutes walking distance)?

1	2	3	4	5
None	A few	Some	Most	All

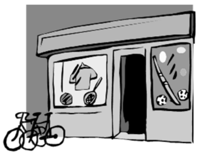

B. Shops, facilities, and other things in your neighbourhood

About how long would it take to get from your home to the nearest businesses or facilities listed below if you walked to them?

Please put a check mark "√" next to your answer (Please only use one check mark "√" for each question).

	1-5 min	6-10 min	11-20 min	20-30 min	30+ min	Do not know
Example: gas station	1. ____	2. ____	3. √	4. ____	5. ____	8. _____
1. convenience/small grocery shop	1. ____	2. ____	3. ___	4. ____	5. ____	8. _____
2. supermarket	1. ____	2. ____	3. ___	4. ____	5. ____	8. _____
3. two-dollar shop	1. ____	2. ____	3. ___	4. ____	5. ____	8. _____
4. fresh food market	1. ____	2. ____	3. ___	4. ____	5. ____	8. _____
5. hardware store (e.g. Bunnings, Masters)	1. ____	2. ____	3. ___	4. ____	5. ____	8. _____
6. clothing & shoes shop	1. ____	2. ____	3. ___	4. ____	5. ____	8. _____
7. pharmacy/drugstore	1. ____	2. ____	3. ___	4. ____	5. ____	8. _____
8. book / stationary shop	1. ____	2. ____	3. ___	4. ____	5. ____	8. _____
9. video / audio shop	1. ____	2. ____	3. ___	4. ____	5. ____	8. _____
10. library	1. ____	2. ____	3. ___	4. ____	5. ____	8. _____
11. laundry/dry cleaners	1. ____	2. ____	3. ___	4. ____	5. ____	8. _____
12. salon/barber shop	1. ____	2. ____	3. ___	4. ____	5. ____	8. _____
13. bank/credit union	1. ____	2. ____	3. ___	4. ____	5. ____	8. _____
14. post office	1. ____	2. ____	3. ___	4. ____	5. ____	8. _____
15. doctor/clinical service	1. ____	2. ____	3. ___	4. ____	5. ____	8. _____
16. primary school	1. ____	2. ____	3. ___	4. ____	5. ____	8. _____
17. nursery schools	1. ____	2. ____	3. ___	4. ____	5. ____	8. _____

About how long would it take to get from your home to the <u>nearest</u> businesses or facilities listed below if you <u>walked</u> to them?

Please put a check mark "√" next to your answer (Please only use <u>one</u> check mark "√" for each question).

	1-5 min	6-10 min	11-20 min	20-30 min	30+ min	Do not know
18. chained Western or Chinese fast-food restaurant (e.g. MacDonald, KFC, Pizza Hut)	1. ____	2. ____	3. ___	4. ____	5. ____	8. _____
19. Chinese coffee shop or noodle shop	1. ____	2. ____	3. ___	4. ____	5. ____	8. _____
20. Chinese non-fast food restaurant	1. ____	2. ____	3. ___	4. ____	5. ____	8. _____
21. Western non-fast food restaurant (sit-down restaurants that employ professional staff or chefs. They are not fast-food restaurant or coffee shops)	1. ____	2. ____	3. ___	4. ____	5. ____	8. _____
22. coffee shop (it sells coffee and snacks)	1. ____	2. ____	3. ___	4. ____	5. ____	8. _____
23. community centre	1. ____	2. ____	3. ___	4. ____	5. ____	8. _____
24. elderly centre	1. ____	2. ____	3. ___	4. ____	5. ____	8. _____
25. park	1. ____	2. ____	3. ___	4. ____	5. ____	8. _____
26. gym or fitness facility	1. ____	2. ____	3. ___	4. ____	5. ____	8. _____
27. swimming pool	1. ____	2. ____	3. ___	4. ____	5. ____	8. _____
28. religious places (e.g. Church, temples)	1. ____	2. ____	3. ___	4. ____	5. ____	8. _____
29. public toilet	1. ____	2. ____	3. ___	4. ____	5. ____	8. _____
30. bakery / cake shop	1. ____	2. ____	3. ___	4. ____	5. ____	8. _____
31. bus stop	1. ____	2. ____	3. ___	4. ____	5. ____	8. _____
32. tram stop	1. ____	2. ____	3. ___	4. ____	5. ____	8. _____
33. train stop	1. ____	2. ____	3. ___	4. ____	5. ____	8. _____
34. lottery/betting outlet	1. ____	2. ____	3. ___	4. ____	5. ____	8. _____
35. place where you can grow vegetables and fruit (e.g. own garden or public garden)	1. ____	2. ____	3. ___	4. ____	5. ____	8. _____

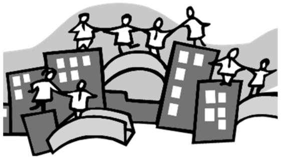

C. Access to services and transportation

Please choose the answer that best applies to you and your neighbourhood.

Please put a check mark "√" next to your answer (Please only use <u>one</u> check mark "√" for each question).

ATTENTION PLEASE:

- *Neighbourhood or easy walking means within 1km or 10-15 minute walking distance from where you live.*
- *The statements below are about Australia and the current situation in your neighbourhood.*
- *"Agree" - means that you agree that the statement is true.*
- *"Disagree" - means that you do not think that the statement is true.*

1. Shops are within easy walking distance of my home (within 1km or 10-15 minute walking distance from where I live).

1	2	3	4	5
strongly disagree	disagree	unsure	agree	strongly agree

2. Shopping areas are easily accessible via public transport.

1	2	3	4	5
strongly disagree	disagree	unsure	agree	strongly agree

3. There are many places to go within walking distance of my home (within 1km or 10-15 minute walking distance).

1	2	3	4	5
strongly disagree	disagree	unsure	agree	strongly agree

4. There are many recreational facilities in my neighbourhood (within 1km or 10-15 minute walking distance).

1	2	3	4	5
strongly disagree	disagree	unsure	agree	strongly agree

5. The recreational facilities in my neighbourhood (within 1km or 10-15 minute walking distance) are affordable. (If you do not have any recreational facilities in your neighbourhood, please use "Not applicable")

1	2	3	4	5	9
strongly disagree	disagree	unsure	agree	strongly agree	Not applicable

6. Recreational centres in my neighbourhood (within 1km or 10-15 minute walking distance) provide a variety of activities for the elderly. (If you don't have any recreational centres in your neighbourhood, please use "Not applicable")

1	2	3	4	5	9
strongly disagree	disagree	unsure	agree	strongly agree	Not applicable

7. Recreational centres in my neighbourhood (within 1km or 10-15 minute walking distance) have Chinese-speaking instructors and volunteers. (If you don't have any recreational centres in your neighbourhood, please use "Not applicable".)

1	2	3	4	5	9
strongly disagree	disagree	unsure	agree	strongly agree	Not applicable

8. It is easy to walk to the following transit stop from my home (within 1km or 10-15 minute walking distance).

- Bus stop

1	2	3	4	5
strongly disagree	disagree	unsure	agree	strongly agree

- Tram stop

1	2	3	4	5
strongly disagree	disagree	unsure	agree	strongly agree

- Train stop

1	2	3	4	5
strongly disagree	disagree	unsure	agree	strongly agree

9. Parking in my neighbourhood (within 1km or 10-15 minute walking distance) is too expensive.

1	2	3	4	5	9
strongly disagree	disagree	unsure	agree	strongly agree	Not applicable

10. Public transport in my neighbourhood (within 1km or 10-15 minute walking distance) is easy to use. (e.g. easy to swipe Myki, not crowded or easy to get on and off.)

- Bus/bus stop

1	2	3	4	5	9
strongly disagree	disagree	unsure	agree	strongly agree	Not applicable

- Tram/tram stop

1	2	3	4	5	9
strongly disagree	disagree	unsure	agree	strongly agree	Not applicable

- Train/train stop

1	2	3	4	5	9
strongly disagree	disagree	unsure	agree	strongly agree	Not applicable

11. Public transport in my neighbourhood (within 1km or 10-15 minute walking distance) provides clear information on the next stop (stop name or number) so that I know when I need to get off. (If you do not have any public transports in your neighbourhood, please use “Not applicable”.)

- Bus

1	2	3	4	5	9
strongly disagree	disagree	unsure	agree	strongly agree	Not applicable

- Tram

1	2	3	4	5	9
strongly disagree	disagree	unsure	agree	strongly agree	Not applicable

- Train

1	2	3	4	5	9
strongly disagree	disagree	unsure	agree	strongly agree	Not applicable

12. Public transport in my neighbourhood (within 10-15 or 1km walking distance) is frequent. (If you do not have a specific type of public transport in your neighbourhood, please use "Not applicable".)

- Bus

1	2	3	4	5	9
strongly disagree	disagree	unsure	agree	strongly agree	Not applicable

- Tram

1	2	3	4	5	9
strongly disagree	disagree	unsure	agree	strongly agree	Not applicable

- Train

1	2	3	4	5	9
strongly disagree	disagree	unsure	agree	strongly agree	Not applicable

13. Public transport services are too expensive.

1	2	3	4	5
strongly disagree	disagree	unsure	agree	strongly agree

14. Transit stop and public transport signage is clear (e.g. transit stop number or bus number is easy to read).

- Bus

1	2	3	4	5
strongly disagree	disagree	unsure	agree	strongly agree

- Tram

1	2	3	4	5
strongly disagree	disagree	unsure	agree	strongly agree

- Train

1	2	3	4	5
strongly disagree	disagree	unsure	agree	strongly agree

15. There is convenient and affordable public transport to get to/from grocery shops in my neighbourhood (within 1km or 10-15 minute walking distance).

1	2	3	4	5
strongly disagree	disagree	unsure	agree	strongly agree

16. The streets in my neighbourhood (within 1km or 10-15 minute walking distance) are hilly, making my neighbourhood difficult to walk in.

1	2	3	4	5
strongly disagree	disagree	unsure	agree	strongly agree

17. There are major barriers to walking in my neighbourhood (within 1km or 10-15 minute walking distance), which make it hard to get from place to place (for example, freeways, railway lines, rivers, steep staircases, roadwork).

1	2	3	4	5
strongly disagree	disagree	unsure	agree	strongly agree

18. I need to walk over a bridge or through a tunnel to access the nearest services in my neighbourhood (within 10-15 or 1km walking distance).

1	2	3	4	5
strongly disagree	disagree	unsure	agree	strongly agree

19. I can easily access the entrance/exit of the building I live in (e.g. my house is on flat ground therefore it is easy to access the entrance or there is a lift that I can use).

1	2	3	4	5
strongly disagree	disagree	unsure	agree	strongly agree

D. Physical food environment

The next few questions ask about a healthy diet. By this, we mean a diet that includes plenty of fruit and vegetables, and does not contain a lot of fat, salt and added sugar foods.

Please choose the answer that best applies to you and your neighbourhood.

Please put a check mark "√" next to your answer (Please only use one check mark "√" for each question).

ATTENTION PLEASE:

- *Neighbourhood or easy walking means within 1km or 10-15 minute walking distance from where you live.*
- *The statements below are about Australia and the current situation in your neighbourhood.*
- *"Agree" - means that you agree that the statement is true.*
- *"Disagree" - means that you do not think that the statement is true.*

1. There are many grocery shops (including Chinese, Western or from other countries) in my neighbourhood (within 10-15 minute or 1km walking distance).

1	2	3	4	5
strongly disagree	disagree	unsure	agree	strongly agree

2. The food outlets in my neighbourhood (within 10-15 minute or 1km walking distance) implement strict food and hygiene policies. (If you don't have any food outlets in your neighbourhood, please choose "Not applicable")

1	2	3	4	5	9
strongly disagree	disagree	unsure	agree	strongly agree	Not applicable

3. Healthy foods cost too much (e.g., vegetables, fruit, foods that do not contain a lot of fat, salt or added sugar)

1	2	3	4	5
strongly disagree	disagree	unsure	agree	strongly agree

4. There are some food shops in my neighbourhood (within 10-15 minute or 1km walking distance) that have an organic food section (Organic food means produce grown without using synthetic pesticides or artificial fertilisers and are not genetically modified). (If you do not have any food shop in your neighbourhood, please chose "Not applicable")"

1	2	3	4	5	9
strongly disagree	disagree	unsure	agree	strongly agree	Not applicable

5. There are shops that provide a large selection of low-fat products in my neighbourhood (within 10-15 minute or 1km walking distance). (If you don't have any shops in your neighbourhood, please choose "Not applicable"

1	2	3	4	5	9
strongly disagree	disagree	unsure	agree	strongly agree	Not applicable

6. There are shops that sell high quality, fresh fruit and vegetables in my neighbourhood (within 10-15 minute or 1km walking distance). (If you don't have any shops in your neighbourhood, please choose "Not applicable")

1	2	3	4	5	9
strongly disagree	disagree	unsure	agree	strongly agree	Not applicable

7. The shops (including Chinese and Western shops) in my neighbourhood (within 10-15 minute or 1km walking distance) sell foods that have Chinese food labelling indicating whether a product is a healthy option. (If you don't have any shops in your neighbourhood, please choose "Not applicable")

1	2	3	4	5	9
strongly disagree	disagree	unsure	agree	strongly agree	Not applicable

8. There is an Asian grocery shop that I can go to in my neighbourhood (within 10-15 minute or 1km walking distance).

1	2	3	4	5
strongly disagree	disagree	unsure	agree	strongly agree

9. Residents in my neighbourhood (within 10-15mins or 1km walking distance) can grow vegetables in their own garden.

1	2	3	4	5
strongly disagree	disagree	unsure	agree	strongly agree

10. There are shops that sale fresh fruit and vegetable juice in my neighbourhood (within 10-15mins or 1km walking distance). (If you do not have any shops in your neighbourhood, please choose "Not applicable"

1	2	3	4	5	9
strongly disagree	disagree	unsure	agree	strongly agree	Not applicable

E. Destinations for socialising

Please choose the answer that best applies to you and your neighbourhood.

Please put a check mark "√" next to your answer (Please only use one check mark "√" for each question).

ATTENTION PLEASE:

- *Neighbourhood or easy walking means within 1km or 10-15 minute walking distance from where you live.*
- *The statements below are about Australia and the current situation in your neighbourhood.*
- *"Agree" - means that you agree that the statement is true.*
- *"Disagree" - means that you do not think that the statement is true.*

1. There is a Chinese senior Club in my neighbourhood (within 10-15 minute or 1km walking distance).

1	2	3	4	5
strongly disagree	disagree	unsure	agree	strongly agree

2. My neighbourhood (within 1km or 10-15 minutes walking distance) provides public housing for elderly.

1	2	3	4	5
strongly disagree	disagree	unsure	agree	strongly agree

3. There are many destinations and services where people can meet in my neighbourhood (within 1km or 10-15 minutes walking distance).

1	2	3	4	5
strongly disagree	disagree	unsure	agree	strongly agree

4. There is a library that provides Chinese books/magazines in my neighbourhood (within 1km or 10-15 minutes walking distance), providing a convenience meeting point.

1	2	3	4	5
strongly disagree	disagree	unsure	agree	strongly agree

5. There is free Wi-Fi in my neighbourhood (e.g., in a library or restaurant within 1km or 10-15 minutes walking distance) that makes it possible for me to keep in touch with my friends and family.

1	2	3	4	5
strongly disagree	disagree	unsure	agree	strongly agree

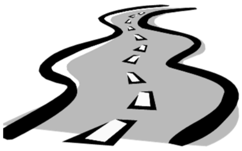

F. Streets in my neighbourhood

Please choose the answer that best applies to you and your neighbourhood.

Please put a check mark "√" next to your answer (Please only use <u>one</u> check mark "√" for each question).

ATTENTION PLEASE:

- *Neighbourhood or easy walking means within 1km or 10-15 minute walking distance from where you live.*
- *The statements below are about Australia and the current situation in your neighbourhood.*
- *"Agree" - means that you agree that the statement is true.*
- *"Disagree" - means that you do not think that the statement is true.*

1. The streets in my neighbourhood (within 1km or 10-15 minutes walking distance) have many cul-de-sacs (dead-end streets).

1	2	3	4	5
strongly disagree	disagree	unsure	agree	strongly agree

2. The distance between intersections in my neighbourhood (within 1km or 10-15 minutes walking distance) is usually short.

1	2	3	4	5
strongly disagree	disagree	unsure	agree	strongly agree

3. There are many alternative routes for getting from place to place in my neighbourhood (within 1km or 10-15 minutes walking distance). (I don't have to go the same way every time.)

1	2	3	4	5
strongly disagree	disagree	unsure	agree	strongly agree

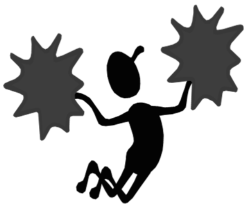

You're making great progress......keep it up!

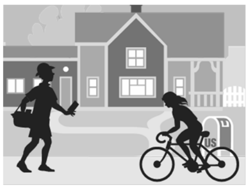

G. Places for walking

Please choose the answer that best applies to you and your neighbourhood.

Please put a check mark "√" next to your answer (Please only use <u>one</u> check mark "√" for each question).

ATTENTION PLEASE:

- *Neighbourhood or easy walking means within 1km or 10-15 minute walking distance from where you live.*
- *The statements below are about Australia and the current situation in your neighbourhood.*
- *"Agree" - means that you agree that the statement is true.*
- *"Disagree" - means that you do not think that the statement is true.*

1. There are sidewalks on most of the streets in my neighbourhood (within 1km or 10-15 minutes walking distance).

1	2	3	4	5
strongly disagree	disagree	unsure	agree	strongly agree

2. There are motor vehicles parked on the sidewalks in my neighbourhood (within 1km or 10-15 minutes walking distance) making it difficult to walk.

1	2	3	4	5
strongly disagree	disagree	unsure	agree	strongly agree

3. There is a fence or grass/dirt strip that separates the streets from the sidewalks in my neighbourhood (within 1km or 10-15 minutes walking distance).

1	2	3	4	5
strongly disagree	disagree	unsure	agree	strongly agree

4. The streets in my neighbourhood (within 1km or 10-15 minutes walking distance) are well lit at night.

1	2	3	4	5
strongly disagree	disagree	unsure	agree	strongly agree

5. There are crosswalks and pedestrian signals to help walkers to cross busy streets in my neighbourhood (within 1km or 10-15 minutes walking distance).

1	2	3	4	5
strongly disagree	disagree	unsure	agree	strongly agree

6. There are many covered sidewalks in my neighbourhood (within 1km or 10-15 minutes walking distance).

1	2	3	4	5
strongly disagree	disagree	unsure	agree	strongly agree

7. There are indoor, air-conditioned places (shopping malls) in my neighbourhood (within 1km or 10-15 minutes walking distance) where people can walk.

1	2	3	4	5
strongly disagree	disagree	unsure	agree	strongly agree

8. The streets and sidewalks in my neighbourhood (within 1km or 10-15 minutes walking distance) are often slippery (e.g., when it rains, the floor becomes slippery, or people often throw water on the street).

1	2	3	4	5
strongly disagree	disagree	unsure	agree	strongly agree

9. There are sitting facilities (e.g., benches) where I can rest in my neighbourhood (within 1km or 10-15 minutes walking distance).

1	2	3	4	5
strongly disagree	disagree	unsure	agree	strongly agree

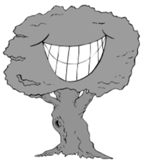

H. Neighbourhood surroundings

Please choose the answer that best applies to you and your neighbourhood.

Please put a check mark “V” next to your answer (Please only use one check mark “v” for each question).

ATTENTION PLEASE:

- *Neighbourhood or easy walking means within 1km or 10-15 minute walking distance from where you live.*
- *The statements below are about Australia and the current situation in your neighbourhood.*
- *“Agree” - means that you agree that the statement is true.*
- *“Disagree” - means that you do not think that the statement is true.*

1. There are trees along the streets in my neighbourhood (within 1km or 10-15 minutes walking distance).

1	2	3	4	5
strongly disagree	disagree	unsure	agree	strongly agree

2. There are many interesting things to look at while walking in my neighbourhood (within 1km or 10-15 minutes walking distance).

1	2	3	4	5
strongly disagree	disagree	unsure	agree	strongly agree

3. There are many attractive natural sights in my neighbourhood (within 1km or 10-15 minutes walking distance), such as landscaping and views.

1	2	3	4	5
strongly disagree	disagree	unsure	agree	strongly agree

4. There are attractive buildings/homes in my neighbourhood (within 1km or 10-15 minutes walking distance).

1	2	3	4	5
strongly disagree	disagree	unsure	agree	strongly agree

5. The level of air pollution in my neighbourhood (within 1km or 10-15 minutes walking distance) is often high.

1	2	3	4	5
strongly disagree	disagree	unsure	agree	strongly agree

6. There are lots of animal droppings in my neighbourhood (within 1km or 10-15 minutes walking distance).

1	2	3	4	5
strongly disagree	disagree	unsure	agree	strongly agree

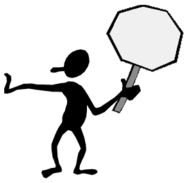

I. Traffic hazards

Please choose the answer that best applies to you and your neighbourhood.

Please put a check mark "V" next to your answer (Please only use <u>one</u> check mark "V" for each question).

ATTENTION PLEASE:

- *Neighbourhood or easy walking means within 1km or 10-15 minute walking distance from where you live.*
- *The statements below are about Australia and the current situation in your neighbourhood.*
- *"Agree" - means that you agree that the statement is true.*
- *"Disagree" - means that you do not think that the statement is true.*

1. There is so much traffic along <u>nearby</u> streets in my neighbourhood (within 1km or 10-15 minutes walking distance), which makes it difficult or unpleasant to walk.

1	2	3	4	5
strongly disagree	disagree	unsure	agree	strongly agree

2. The speed of traffic on most <u>nearby</u> streets in my neighbourhood (within 1km or 10-15 minutes walking distance) is usually slow (40 km/h or less).

1	2	3	4	5
strongly disagree	disagree	unsure	agree	strongly agree

3. Most drivers exceed the posted speed limits while driving in my neighbourhood (within 1km or 10-15 minutes walking distance).

1	2	3	4	5
strongly disagree	disagree	unsure	agree	strongly agree

4. There are parked vehicles in my neighbourhood (within 1km or 10-15 minutes walking distance) that block my vision and make it difficult to safely cross the road.

1	2	3	4	5
strongly disagree	disagree	unsure	agree	strongly agree

5. The streets in my neighbourhood (within 1km or 10-15 minutes walking distance) have many passing cars, which make me afraid to cross the road.

1	2	3	4	5
strongly disagree	disagree	unsure	agree	strongly agree

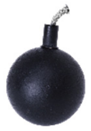

J. Safety from crime

Please choose the answer that best applies to you and your neighbourhood.

Please put a check mark "√" next to your answer (Please only use <u>one</u> check mark "√" for each question).

ATTENTION PLEASE:

- *Neighbourhood or easy walking means within 1km or 10-15 minute walking distance from where you live.*
- *The statements below are about Australia and the current situation in your neighbourhood.*
- *"Agree" - means that you agree that the statement is true.*
- *"Disagree" - means that you do not think that the statement is true.*

1. Walkers on the streets in my neighbourhood (within 1km or 10-15 minutes walking distance) can be easily seen by other people.

1	2	3	4	5
strongly disagree	disagree	unsure	agree	strongly agree

2. The crime rate in my neighbourhood (within 1km or 10-15 minutes walking distance) is high.

1	2	3	4	5
strongly disagree	disagree	unsure	agree	strongly agree

3. The crime rate in my neighbourhood (within 1km or 10-15 minutes walking distance) makes it unsafe to go on walks <u>during the day</u>.

1	2	3	4	5
strongly disagree	disagree	unsure	agree	strongly agree

4. The crime rate in my neighbourhood (within 1km or 10-15 minutes walking distance) makes it unsafe to go on walks <u>at night</u>.

1	2	3	4	5
strongly disagree	disagree	unsure	agree	strongly agree

5. There are many homeless people, drug addicts and/or prostitutes in my neighbourhood (within 1km or 10-15 minutes walking distance).

1	2	3	4	5
strongly disagree	disagree	unsure	agree	strongly agree

6. It would be difficult to ask for help in my neighbourhood (within 1km or 10-15 minutes walking distance) because there are not many people around.

1	2	3	4	5
strongly disagree	disagree	unsure	agree	strongly agree

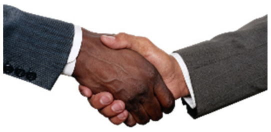

K. Social environment and communication

Please choose the answer that best applies to you and your neighbourhood.

Please put a check mark "V" next to your answer (Please only use <u>one</u> check mark "√" for each question).

ATTENTION PLEASE:

- *Neighbourhood or easy walking means within 1km or 10-15 minute walking distance from where you live.*
- *The statements below are about Australia and the current situation in your neighbourhood.*
- *"Agree" - means that you agree that the statement is true.*
- *"Disagree" - means that you do not think that the statement is true.*

1. Volunteer organisations in my neighbourhood (within 1km or 10-15 minutes walking distance) organise physical activity sessions (e.g., walking activities) (if you do not have any volunteer organisations in your neighbourhood, please use "Not applicable").

1	2	3	4	5	9
strongly disagree	disagree	unsure	agree	strongly agree	Not applicable

2. I can easily find information in Chinese on health, community policies and community services promoting physical activity in my neighbourhood (within 1km or 10-15 minutes walking distance).

1	2	3	4	5
strongly disagree	disagree	unsure	agree	strongly agree

3. There are people (anyone, including family, friends, neighbours, professionals, etc.) in my neighbourhood (within 1km or 10-15 minutes walking distance) who I can talk to about healthy eating.

1	2	3	4	5
strongly disagree	disagree	unsure	agree	strongly agree

4. Our Australian local Chinese newspaper / magazine publishes information on healthy eating.

1	2	3	4	5
strongly disagree	disagree	unsure	agree	strongly agree

5. Our neighbourhood (within 1km or 10-15 minutes walking distance) organises talks on healthy eating in Chinese.

1	2	3	4	5
strongly disagree	disagree	unsure	agree	strongly agree

6. Clinics, shops and other services in my neighbourhood (within 1km or 10-15 minutes walking distance) have staff who can speak English and Chinese (if you do not have clinics, shops or other services in your neighbourhood please use "Not applicable").

1	2	3	4	5	9
strongly disagree	disagree	unsure	agree	strongly agree	Not applicable

7. Volunteers organise free-of-charge group activities for Chinese elders in our neighbourhood (within 1km or 10-15 minutes walking distance).

1	2	3	4	5
strongly disagree	disagree	unsure	agree	strongly agree

8. My neighbourhood (within 1km or 10-15 minutes walking distance) provides formal or informal opportunities for Chinese- and English-speaking older residents to network.

1	2	3	4	5
strongly disagree	disagree	unsure	agree	strongly agree

9. There are many Chinese-speaking residents in my neighbourhood (within 1km or 10-15 minutes walking distance).

1	2	3	4	5
strongly disagree	disagree	unsure	agree	strongly agree

10. There are Chinese-speaking policemen in my neighbourhood (within 1km or 10-15 minutes walking distance).

1	2	3	4	5
strongly disagree	disagree	unsure	agree	strongly agree

Thank you so much for your time and participation!

Questionnaire: Perceived barriers to engaging in health-enhancing behaviours

A. Perceived barriers to physical activity

This section is about regular exercise, including walking for recreational purposes (e.g., walking in leisure time, Tai-Chi, etc.).

Do the following reasons prevent you from getting regular exercise including walking for recreational purposes (e.g., walking in leisure time, Tai-Chi, etc.)?
Please circle the most appropriate answer – one response per question.

Example: "Fear of injury" [often] prevents me from getting regular exercise including walking for recreational purposes.

**Never**	**Rarely**	**Sometimes**	**Often**	**Very often**
1	2	3	4 (circled)	5

1. "Self-conscious about my looks" (e.g. dislike being sweaty or worry about own weight) ______ prevents me from getting regular exercise including walking for recreational purposes.

**Never**	**Rarely**	**Sometimes**	**Often**	**Very often**
1	2	3	4	5

2. "Lack of interest in physical activity" ______prevents me from getting regular exercise including walking for recreational purposes.

**Never**	**Rarely**	**Sometimes**	**Often**	**Very often**
1	2	3	4	5

3. "Lack of self-discipline" (e.g., feeling lazy and do not want to do the pre-planned physical activity) ______prevents me from getting regular exercise including walking for recreational purposes.

**Never**	**Rarely**	**Sometimes**	**Often**	**Very often**
1	2	3	4	5

4. "Lack of time" ______prevents me from getting regular exercise including walking for recreational purposes.

**Never**	**Rarely**	**Sometimes**	**Often**	**Very often**
1	2	3	4	5

5. "Lack of energy" ______prevents me from getting regular exercise including walking for recreational purposes.

**Never**	**Rarely**	**Sometimes**	**Often**	**Very often**	
1	2	3	4	5	9

6. "Lack of company" ______prevents me from getting regular exercise including walking for recreational purposes.

**Never**	**Rarely**	**Sometimes**	**Often**	**Very often**
1	2	3	4	5

7. "Lack of enjoyment in physical activity" ______prevents me from getting regular exercise including walking for recreational purposes.

**Never**	**Rarely**	**Sometimes**	**Often**	**Very often**
1	2	3	4	5

8. "Discouragement by others" ______prevents me from getting regular exercise including walking for recreational purposes.

**Never**	**Rarely**	**Sometimes**	**Often**	**Very often**
1	2	3	4	5

9. "Lack of equipment" ______prevents me from getting regular exercise including walking for recreational purposes.

**Never**	**Rarely**	**Sometimes**	**Often**	**Very often**
1	2	3	4	5

10. "Lack of good weather (e.g. too hot or too wet)" ______prevents me from getting regular exercise including walking for recreational purposes.

**Never**	**Rarely**	**Sometimes**	**Often**	**Very often**
1	2	3	4	5

11. "Lack of skills/knowledge to do physical activity" ______prevents me from getting regular exercise including walking for recreational purposes.

**Never**	**Rarely**	**Sometimes**	**Often**	**Very often**
1	2	3	4	5

12. "Lack of facilities, community activities for Chinese, space or places to walk" ______prevent me from getting regular exercise including walking for recreational purposes.

**Never**	**Rarely**	**Sometimes**	**Often**	**Very often**
1	2	3	4	5

13. "Lack of good health" ______prevents me from getting regular exercise including walking for recreational purposes.

**Never**	**Rarely**	**Sometimes**	**Often**	**Very often**
1	2	3	4	5

14. “Fear of injury” ______ prevents me from getting regular exercise including walking for recreational purposes.

Never	Rarely	Sometimes	Often	Very often
1	2	3	4	5

15. “Air pollution in Australia” ______ prevents me from getting regular exercise including walking for recreational purposes.

Never	Rarely	Sometimes	Often	Very often
1	2	3	4	5

16. “Lack of money” ______ prevents me from getting regular exercise including walking for recreational purposes.

Never	Rarely	Sometimes	Often	Very often
1	2	3	4	5

17. “Inability to communicate with others in English” ______ prevents me from getting regular exercise including walking for recreational purposes.

Never	Rarely	Sometimes	Often	Very often
1	2	3	4	5

18. “Lack of information on community activities” ______ prevents me from getting regular exercise including walking for recreational purposes.

Never	Rarely	Sometimes	Often	Very often
1	2	3	4	5

19. “Difficulties in getting to/from a recreational destination (e.g. infrequent public transport)” ______ prevents me from getting regular exercise including walking for recreational purposes.

Never	Rarely	Sometimes	Often	Very often
1	2	3	4	5

20. “Being dependent on other family members and lacking freedom” ______ prevents me from getting regular exercise including walking for recreational purposes.

Never	Rarely	Sometimes	Often	Very often
1	2	3	4	5

This section is about regular walking for transport to go to/from places (e.g., walking to go to work or to get groceries).

Do the following reasons prevent you from walking for transport to go to/from places (e.g., walking to go to work or to get groceries)?

Please circle the most appropriate answer – one response per question.

Example: "Lack of energy" [sometimes**]** prevents me from walking for transport to go to/from places.

**Never**	**Rarely**	**Sometimes**	**Often**	**Very often**
1	2	3	4	5

1. "Traffic hazards (e.g., nearby streets have lots of traffic or lack pedestrian crossings)" ______prevent me from walking for transport to go to/from places.

**Never**	**Rarely**	**Sometimes**	**Often**	**Very often**
1	2	3	4	5

2. "Lack of enjoyment in walking" ______prevents me from walking for transport to go to/from places.

**Never**	**Rarely**	**Sometimes**	**Often**	**Very often**
1	2	3	4	5

3. "Physical barriers to destinations (e.g., steep hills)" ______prevent me walking for transport to go to/from places.

**Never**	**Rarely**	**Sometimes**	**Often**	**Very often**
1	2	3	4	5

4. "Lack of time" ______prevents me from walking for transport to go to/from places.

**Never**	**Rarely**	**Sometimes**	**Often**	**Very often**
1	2	3	4	5

5. "Lack of energy" ______prevents me from walking for transport to go to/from places.

**Never**	**Rarely**	**Sometimes**	**Often**	**Very often**
1	2	3	4	5

6. "Lack of company" ______prevents me from walking for transport to go to/from places.

**Never**	**Rarely**	**Sometimes**	**Often**	**Very often**
1	2	3	4	5

7. "Air pollution in Australia" ______prevents me from walking for transport to go to/from places.

**Never**	**Rarely**	**Sometimes**	**Often**	**Very often**
1	2	3	4	5

8. "Discouragement by others" ______prevents me from walking for transport to go to/from places.

**Never**	**Rarely**	**Sometimes**	**Often**	**Very often**
1	2	3	4	5

9. "Lack of good weather" (e.g., too hot; too wet) ______prevents me from walking for transport to go to/from places.

**Never**	**Rarely**	**Sometimes**	**Often**	**Very often**
1	2	3	4	5

10. "Lack of places to walk to" ______prevents me from walking for transport to go to/from places.

Never	Rarely	Sometimes	Often	Very often
1	2	3	4	5

11. "Lack of good health" ______prevents me from walking for transport to go to/from places.

Never	Rarely	Sometimes	Often	Very often
1	2	3	4	5

12. "Crime rate" ______prevents me from walking for transport to go to/from places.

Never	Rarely	Sometimes	Often	Very often
1	2	3	4	5

B. Perceived barriers to healthy eating

Do the following reasons prevent you from eating a healthy diet (e.g., vegetables, fruits, foods that do not contain a lot of fat, salt and added sugar)?

Please circle the most appropriate answer – one response per question.

Example: "Grocery shops being too far away from home" [very often] prevents me from eating a healthy diet.

Never	Rarely	Sometimes	Often	Very often
1	2	3	4	5

1. "Lack of information about a healthy diet in Chinese" (e.g. lack of Chinese newspaper, website, etc.) ______prevents me from eating a healthy diet.

Never	Rarely	Sometimes	Often	Very often
1	2	3	4	5

2. "Not enjoying eating healthy foods (e.g. vegetables, fruit, foods that do not contain a lot of fat, salt and added sugar)" ______prevents me from eating a healthy diet.

Never	Rarely	Sometimes	Often	Very often
1	2	3	4	5

3. "Healthy foods (e.g. vegetables, fruit, foods that do not contain a lot of fat, salt and added sugar) being too expensive" ______prevents me from eating a healthy diet.

Never	Rarely	Sometimes	Often	Very often
1	2	3	4	5

4. "Lack of time to prepare healthy foods (e.g. vegetables, fruit, foods that do not contain a lot of fat, salt and added sugar)"______prevents me from eating a healthy diet. (If you do not cook, please choose "Not applicable").

Never	Rarely	Sometimes	Often	Very often	Not applicable
1	2	3	4	5	9

5. "Lack of energy/motivation to prepare healthy foods (e.g. vegetables, fruit, foods that do not contain a lot of fat, salt and added sugar)" ______prevents me from eating a healthy diet. (If you do not cook, please choose "Not applicable").

**Never**	**Rarely**	**Sometimes**	**Often**	**Very often**	**Not applicable**
1	2	3	4	5	9

6. "Lack of skills to plan, shop for, prepare or cook healthy foods (e.g. vegetables, fruit, foods that do not contain a lot of fat, salt and added sugar)" ______prevents me from eating a healthy diet. (If you do not cook, please choose "Not applicable").

**Never**	**Rarely**	**Sometimes**	**Often**	**Very often**	**Not applicable**
1	2	3	4	5	9

7. "Grocery shops being too far away from home" ______prevents me from eating a healthy diet.

**Never**	**Rarely**	**Sometimes**	**Often**	**Very often**
1	2	3	4	5

8. "Eating out" ______prevents me from eating a healthy diet (e.g., If when I eat out, I always choose healthy food options, then "eating out" never prevents me from eating a healthy diet)

**Never**	**Rarely**	**Sometimes**	**Often**	**Very often**
1	2	3	4	5

9. "Lack of healthy options in food shops (e.g. vegetables, fruit, foods that do not contain a lot of fat, salt and added sugar)" ______prevents me from eating a healthy diet.

**Never**	**Rarely**	**Sometimes**	**Often**	**Very often**
1	2	3	4	5

10. "Lack of friends' support for eating a healthy diet" ______prevents me from eating a healthy diet. (If you do not have friends, please choose "Not applicable".)

**Never**	**Rarely**	**Sometimes**	**Often**	**Very often**	**Not applicable**
1	2	3	4	5	9

11. "Lack of partner's support for eating a healthy diet" ______prevents me from eating a healthy diet. (If you do not have a partner, please choose "Not applicable".)

**Never**	**Rarely**	**Sometimes**	**Often**	**Very often**	**Not applicable**
1	2	3	4	5	9

12. "Lack of children's support for eating a healthy diet" ______prevents me from eating a healthy diet?. (If you do not have children, please choose "Not applicable".)

**Never**	**Rarely**	**Sometimes**	**Often**	**Very often**	**Not applicable**
1	2	3	4	5	9

C. Perceived barriers to socialising

Do the following reasons prevent you from socialising?
Please circle the most appropriate answer – one response per question.

Example: "Lack of Chinese-speaking people" [very often**]** prevents me from socialising.

Never	Rarely	Sometimes	Often	Very often
1	2	3	4	5

1. "Inability to speak English" ______ prevents me from socialising.

Never	Rarely	Sometimes	Often	Very often
1	2	3	4	5

2. "Lack of activity centres and facilities for Chinese elderly" ______ prevents me from socialising.

Never	Rarely	Sometimes	Often	Very often
1	2	3	4	5

3. "Lack of good health" ______ prevents me from socialising.

Never	Rarely	Sometimes	Often	Very often
1	2	3	4	5

4. "Poor public transport" ______ prevents me from socialising.

Never	Rarely	Sometimes	Often	Very often
1	2	3	4	5

5. "Shyness" ______ prevents me from socialising.

Never	Rarely	Sometimes	Often	Very often
1	2	3	4	5

6. "Lack of Chinese-speaking people" ______ prevents me from socialising.

Never	Rarely	Sometimes	Often	Very often
1	2	3	4	5

7. "Lack of time" ______ prevents me from socialising.

Never	Rarely	Sometimes	Often	Very often
1	2	3	4	5

8. "Lack of motivation in socialising" ______ prevents me from socialising.

Never	Rarely	Sometimes	Often	Very often
1	2	3	4	5

9. "Lack of information on events, meeting groups for Chinese elderly" ______ prevents me from socialising.

Never	Rarely	Sometimes	Often	Very often
1	2	3	4	5

10. “Lack of partner’s support” ______ prevents me from socialising. (If you do not have a partner, please use “Not applicable”.)

Never	Rarely	Sometimes	Often	Very often	Not applicable
1	2	3	4	5	9

11. “Lack of children’s support” ______ prevents me from socialising. (If you do not have children, please use “Not applicable”.)

Never	Rarely	Sometimes	Often	Very often	Not applicable
1	2	3	4	5	9

Lastly, please write down today’s date _____(month)_______(day)!

Thank you so much for your time and participation!!
